# Engineering the oleaginous yeast *Candida tropicalis* for α-humulene overproduction

**DOI:** 10.1186/s13068-022-02160-8

**Published:** 2022-05-26

**Authors:** Lihua Zhang, Haiquan Yang, Yuanyuan Xia, Wei Shen, Liming Liu, Qi Li, Xianzhong Chen

**Affiliations:** Key Laboratory of Industrial Biotechnology, Ministry of Education, and School of Biotechnology, School of Biotechnology, Jiangnan University, 1800 Lihu Road, Wuxi, 214122 People’s Republic of China

**Keywords:** *Candida tropicalis*, α-Humulene, Rate-limiting enzymes, Metabolic engineering, Mevalonate pathway

## Abstract

**Background:**

α-Humulene is a plant-derived monocyclic sesquiterpenoid with multiple pharmacological activities, and far-reaching potential for the development of new drugs. Currently, the production of α-humulene is typically achieved via plant extraction, which is not sustainable and limited by low yields. The oleaginous yeast *Candida tropicalis* has recently emerged as a valuable host for producing high-value-added chemicals. However, the potential of *C. tropicalis* for terpenoid production has not been exploited.

**Results:**

In this study, *C. tropicalis* was engineered for de novo synthesis of α-humulene from glucose. To improve α-humulene production, the codon-optimised α-humulene synthase gene and the entire endogenous farnesyl diphosphate synthesis pathway were co-overexpressed. Furthermore, bottlenecks in the α-humulene synthase pathway were identified and relieved by overexpressing α-humulene synthase, acetoacetyl-CoA thiolase and NADH-dependent HMG-CoA reductase. Combined with fermentation medium optimisation, the engineered strain produced 195.31 mg/L of α-humulene in shake flasks and 4115.42 mg/L in a bioreactor through fed-batch fermentation, a 253- and 5345-fold increase over the initial production, respectively.

**Conclusions:**

This study demonstrates the potential of *C. tropicalis* for α-humulene production, and presents a platform for the biosynthesis of other terpenoids.

**Supplementary Information:**

The online version contains supplementary material available at 10.1186/s13068-022-02160-8.

## Background

Terpenoids, the largest family of natural compounds (> 55,000 members), are widely used in the fields of food processing, agriculture, medicine and industry [1]. α-Humulene is a highly valued monocyclic sesquiterpenoid generally found in plants associated with its analogues β-humulene and isocaryophyllene [2]. α-Humulene and its isomers possess anti-inflammatory, antimicrobial and antitumour activities [2, 3]. Additionally, α-humulene has an 11-membered-ring and is a key intermediate for chemosynthesis or biosynthesis of zerumbone and other bioactive compounds [4, 5]. Currently, the large-scale production of α-humulene is typically achieved via plant extraction, while the α-humulene content in plants is low and significant differences among varieties and regions [6, 7]. Considering the complicated process and depletion of natural resources, the traditional methods are limited by low yields (only 6.2 g of α-humulene per kilogram of dried unopened flower buds of *Syzygium aromaticum* [6]), and shortage of raw materials. On the other hand, the chemical synthesis of α-humulene suffers from the numerous steps using environmental hazardous catalysts [8]. Therefore, biotechnological process is expected to provide an environmental-friendly and economical alternative.

Recently, α-humulene has been produced by various hosts. Krieg et al. constructed an α-humulene-producing strain of *Cupriavidus necator*, which expressed the α-humulene synthase gene (*ZSS1*) from *Zingiber zerumbet* Smith and overexpressed the heterologous mevalonate (MVA) pathway from *Methylobacterium extorquens* to improve production [9]. The titre of the engineered strain reached 17 mg/g dry cell weight (DCW) using CO_2_ as carbon source and sunlight as energy source. In another study, *Methylotuvimicrobium alcaliphilum* 20Z was engineered to express *ZSS1* in combination with optimisation of the native methylerythritol phosphate (MEP) pathway, and the strain accumulated 0.75 mg/g DCW of α-humulene from methane [10]. By comparison, engineered *Methylobacterium extorquens* (expressing *ZSS1* in combination with a heterologous mevalonate pathway from *Myxococcus xanthus*) produced up to 1.65 g/L α-humulene in methanol-limited fed-batch fermentation [11].

Although these unconventional substrates (CO_2_, methane, methanol and acetate) are considered promising sustainable carbon sources for industrial biomanufacturing, sugar is currently the dominant raw material. Currently, *Saccharomyces cerevisiae*, *Yarrowia lipolytica* and *Escherichia coli* are the three main chassis hosts for natural products produced from sugars. *E. coli* was engineered to express *ZSS1* and a heterologous mevalonate pathway to improve the titre of α-humulene to 1 g/L in terrific broth containing 0.5 g/L of mevalonolactone or 1 g/L of lithium acetoacetate [12]. In general, the MEP pathway in bacteria has a theoretically higher mass yield, but the MVA pathway typically performs better in terms of precursor supply for terpenoid production [13, 14]. To optimise α-humulene production in *S. cerevisiae*, the α-humulene synthesis enzyme was packaged in peroxisomes combined with cytoplasmic engineering [15]. The engineered strain produced 1.73 g/L of α-humulene in a 5-L bioreactor with glucose as carbon source, the highest titre reported to date for microorganisms. A similar strategy has been used for α-farnesene, isoprene, and squalene overproduction [16–18].

**Fig. 1:**
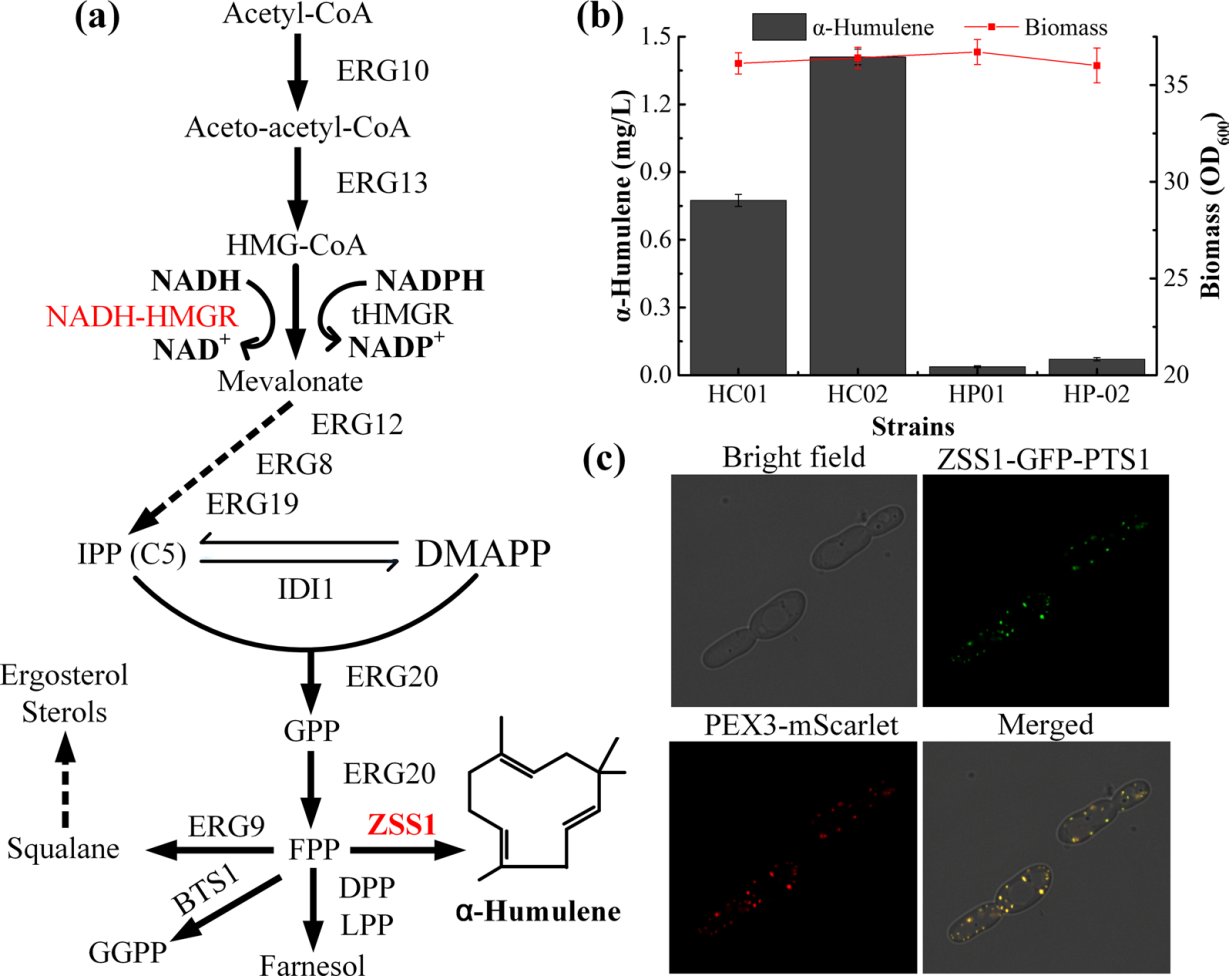
Biosynthesis of α-humulene in *C. tropicalis*. **a** Scheme showing the α-humulene biosynthesis pathway in *C. tropicalis*. ERG10, acetoacetyl-CoA thiolase; ERG13, hydroxymethylglutaryl-CoA synthase; tHMRG, truncated hydroxymethylglutaryl-CoA reductase; NADH-HMGR, NADH-dependent HMG-CoA reductase from *Silicibacter pomeroyi*; ERG12, mevalonate kinase; ERG8, phosphomevalonate kinase; ERG19, mevalonate diphosphate decarboxylase; IDI1, isopentenyl diphosphate isomerase; ERG20, geranyl/farnesyl diphosphate synthase; ZSS1, α-humulene synthase; IPP, isopentenyl diphosphate; DMAPP, dimethylallyl diphosphate; GPP, geranyl diphosphate; FPP, farnesyl diphosphate. **b** α-humulene production by *C. tropicalis* expressing ZSS1 in the cytoplasm (HC01 and HC02) and in peroxisomes (HP01 and HP02). Results are means ± standard deviations of biological triplicates. **c** Fluorescence microscopy of *C. tropicalis* 01 co-expressed the ZSS1-GFP-PTS1 fusion protein and the peroxisome marker PEX3-mScarlet

*Candida tropicalis* is a diploid, oleaginous yeast, and an invaluable host for industrial production due to its robust tolerance to unfavourable conditions [19, 20], and its ability to degrade cyanide [21] and utilise various carbon sources [21, 22]. Moreover, lipid accumulation in this yeast species can reach 58% of dry biomass [23]. It has recently emerged as a valuable host for producing high-value-added chemicals such as long-chain α,ω-dicarboxylic acids [24], ω-hydroxy fatty acids [22] and xylitol [20]. However, inadequate genetic engineering tools for metabolic engineering of complex metabolic pathways have limited the application of *C. tropicalis*. There are few reports on the use of this yeast species in the production of terpenoids. Recently, the CRISPR–Cas9 system for multiple genome editing, pathway assembly [25] and gene interference (CRISPRi, YJ Li et al. unpublished) was developed for *C. tropicalis*. By integrating *carB* (encoding phytoene dehydrogenase) and *carRP* (encoding bi-functional enzymes phytoene synthase and lycopene cyclase) genes from *Mucor circinelloides* into the chromosome of *C. tropicalis*, the resulting DRPB strain could accumulate 0.23 mg/g DCW of β-carotene [25]. This indicates that the terpenoid precursors farnesyl diphosphate (FPP) and geranylgeranyl diphosphate are present in *C. tropicalis* (biosynthesis via the MVA pathway, Fig. 1a), hence the yeast is a potential host for terpenoid production. Acetyl-CoA is a common precursor of lipid biosynthetic pathway and terpenoid pathway. Generally, the oleaginous yeast has a rich acetyl-CoA pool [26]. The oleaginous yeast *Y. lipolytica* has been metabolically engineered for the production of linalool [27], ginsenoside compound K [28], β-carotene [29] and lycopene [30] in recent years. With the development of genetic engineering tools, *C. tropicalis* may become a useful platform strain for terpenoid production.

In this study, we explored the potential of the oleaginous yeast *C. tropicalis* for α-humulene biosynthesis following metabolic engineering. The *ZSS1* gene from *Z. zerumbet* was codon-optimised and integrated into the genome of *C. tropicalis*, and a basal α-humulene-producing strain was constructed. Further improvement in α-humulene production was achieved by overexpressing the entire endogenous FPP synthesis pathway and adjusting gene dosage. Finally, the fermentation conditions for α-humulene production were optimised, and an impressive yield of 4115.42 mg/L of α-humulene was achieved using fed-batch fermentation.

## Results and discussion

### Engineering* C. tropicalis* for α-humulene production

Because *C. tropicalis* lacks an efficient autonomously replicating plasmid, exogenous genes are usually integrated into the genome for stable expression. Previous studies showed that single deletion of *CAT* in *C. tropicalis* had no impact on cells growth [31], therefore the *CtCas9* expression cassette was integrated at the *CAT* locus to generate strain CU-207 for further facilitating genetic manipulation. After the *URA3* marker was excised from *C. tropicalis* CU-207, the resulting uracil auxotrophic strain CU-208 was used as the platform strain for further metabolic engineering. Our previous studies showed that it is challenging to express heterologous genes in *C. tropicalis* without codon optimisation [25, 31]. Thus, the codon-optimised *ZSS1* from *Z. zerumbet* was integrated into the chromosome of *C. tropicalis* CU-208 through CRISPR–Cas9, resulting in strain HC01 (possessing a single copy of *ZSS1* at the *POX5* locus) and HC02 (possessing double copies of *ZSS1* at the *POX5* loci). After 96 h of fermentation, α-humulene production was detected by GC–MS (Fig. 1b and Additional file 2: Fig. S1). HC-02 produced 1.41 mg/L α-humulene, roughly double that of HC-01 (0.77 mg/L), suggesting that the *ZSS1* gene can be successfully expressed in *C. tropicalis*, and the expression level of *ZSS1* might be a key factor influencing α-humulene production. Compared with CU-207, the biomass of HC01 and HC02 was not markedly different, indicating that a low level of α-humulene had little or no effect on yeast growth. Nevertheless, the α-humulene concentration was lower than that reported for *S. cerevisiae* (2.32 mg/L, possessing a single copy of *ZSS1*) [15].

Previous studies have reported that the peroxisome was more appropriate for the synthesis of limonene, α-humulene and squalene in *S. cerevisiae* [15, 17, 32]. Therefore, it is necessary to evaluate whether the peroxisome could be benefit for producing α-humulene in *C. tropicalis*. However, no confirmed peroxisome targeting signals are presented in *C. tropicalis*. Firstly, the function of peroxisome targeting signal-1 (PTS1, SKL) was evaluated with ZSS1-GFP as a reporter. To label peroxisome, a red fluorescence protein was fused with peroxisome membrane protein (PEX3-mScarlet) and co-expressed with ZSS1-GFP-PTS1. Fluorescence microscopy results showed that the GFP and mScarlet signals colocalised (Fig. 1c), indicating that ZSS1-GFP-PTS1 could be transported into peroxisome. However, when α-humulene synthase was directed to peroxisomes by PTS1, only 0.06 mg/L of α-humulene accumulated in the transformant possessing double copies of *ZSS1*-PTS1 (HP02; 0.04 mg/L for HP01 possessing one copy of *ZSS1*-PTS1; Fig. 1b).

### Effects of* HMGR* and* ERG10* overexpression and* ERG9* repression on α-humulene production

The biosynthesis of α-humulene from acetyl-CoA in *C. tropicalis* requires multiple enzymes and complex metabolic regulation (Fig. 1a). Previous studies demonstrated that overexpression of *HMGR* and *ERG10* and repression of *ERG9* expression positively affect terpenoid production in *S. cerevisiae* and *Y. lipolytica* [27, 29, 33]. Therefore, the influence of these three genes on the production of α-humulene in *C. tropicalis* was evaluated.

**Fig. 2:**
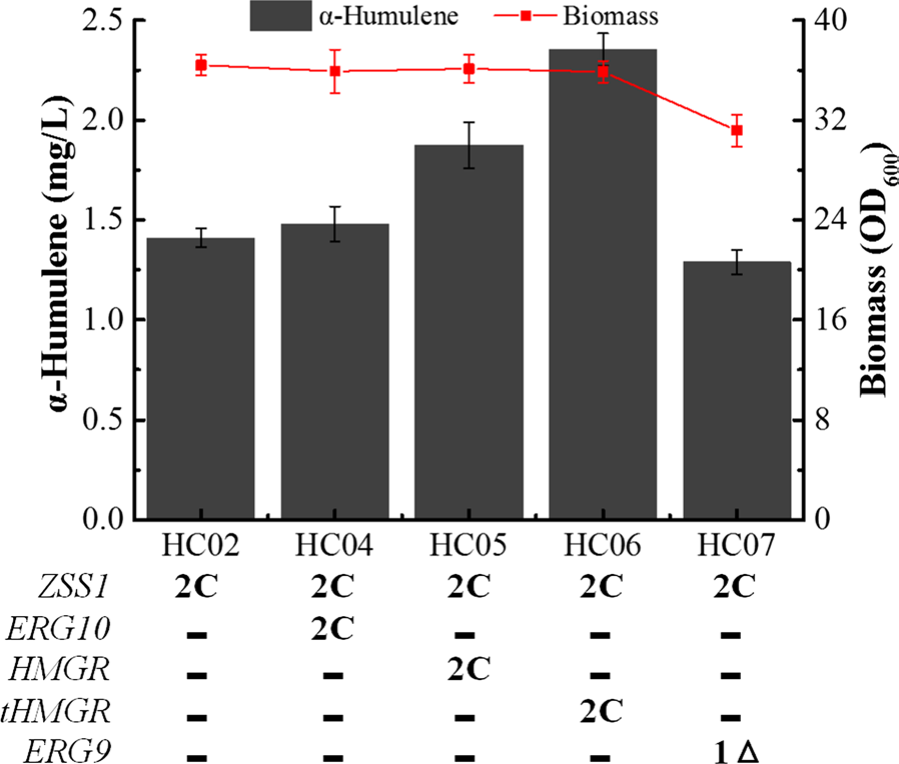
Effects of *ERG10*, *HMGR* and *tHMGR* overexpression and *ERG9* repression in *C. tropicalis* on biomass and α-humulene production: 2C represents the expression of double copies of genes in the cytoplasm and 1Δ represents the disruption one of the *ERG9* alleles. Results are means ± standard deviations of biological triplicates

First, *HMGR* and *ERG10* were expressed in HC03 (a uracil auxotrophic derivative of HC02). The resulting strain HC05 (overexpressing *HMGR*) produced 1.88 mg/L α-humulene, 33.3% more than HC02 (Fig. 2, HC05 vs. HC02). By contrast, overexpressing *ERG10* did not improve production of α-humulene (Fig. 2, HC04 vs. HC02).

It has been reported that the hydroxymethylglutaryl-CoA reductases of *S. cerevisiae* and *Y. lipolytica* share similar structures, with N-terminal multiple transmembrane domains and a C-terminal catalytically active domain [4, 29, 34]. Moreover, the N-terminal domain is a response element for signal regulation; its deletion can enhance protein stability. Thus, a truncated *HMGR* of *C. tropicalis* (*tHMGR*, lacking the N-terminal multiple transmembrane domains, Additional file 2: Fig. S2) was overexpressed in HC03. As expected, α-humulene production in HC06 (overexpressing *tHMGR*) was increased by 66.7% compared to HC02 (Fig. 2).

Squalene synthase (*ERG9*) catalyses the reductive dimerisation of two FPP moieties to form one molecule of squalene. FPP is a precursor of sesquiterpenoids in eukaryotes, while squalene plays an integral role in sterol synthesis (Fig. 1a). In order to increase the FPP flux towards α-humulene biosynthesis, a single copy of *ERG9* was disrupted in HC03, generating strain HC07. Unexpectedly, the biomass and α-humulene titre of HC07 were decreased compared with HC02, although the α-humulene content was improved slightly (Fig. 2). In addition, the content of β-carotene in *ERG9*-disrupted *C. tropicalis* followed the same trend (YJ Li et al. unpublished). RT-qPCR analysis showed that *ERG9* mRNA levels in strain HC07 were 43% lower than in HC02 (Additional file 2: Fig. S3), indicating that expression of *ERG9* was repressed. Similarly, the lycopene production capacity of engineered *Candida utilis* could not be increased when two copies of *ERG9* were deleted in tetraploid yeast [35]. In our previous study, one copy of the *CAT* gene was disrupted in *C. tropicalis*, and carnitine acetyltransferase mRNA levels and enzyme activity were decreased [31]. Meanwhile, the concentration of α,ω-dodecanedioic acid was not changed significantly. These data may indicate that the normal allele can completely (or mostly) cancel the mutant allele in diploid and polyploid yeast.

### Overexpressing the entire α-humulene synthesis pathway to improve α-humulene production

**Fig. 3:**
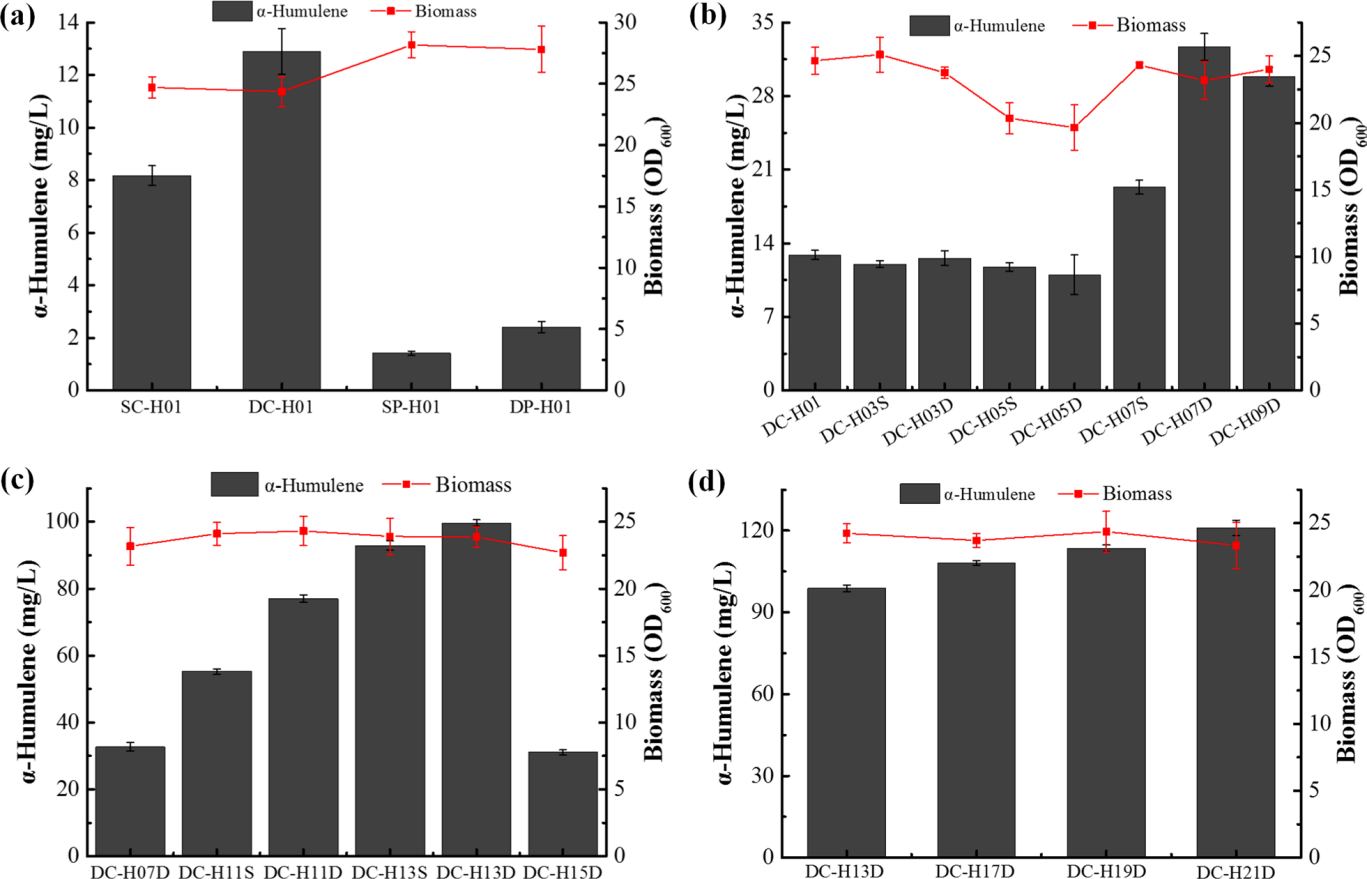
α-Humulene production of engineered strains and identification of the rate-limiting steps of the α-humulene synthesis pathway. **a** Comparison of α-humulene titre between cytoplasm and the peroxisomal pathways. **b** Combined overexpression of α-humulene synthesis pathway genes in DC-H01 to identify bottlenecks in the pathway. **c** Effect of *ZSS1* overexpression on α-humulene production. **d** Effects of t*HMGR*, *NADH-HMGR* and *ERG10* overexpression on α-humulene production. Results are means ± standard deviations of biological triplicates

Although strains HC02 and HP02 could produce α-humulene, production was very low. This might be due to the inefficiency of the native MVA pathway, which is tightly regulated in yeast. Firstly, a short synthetic terminator (*T*_*synth7*_, 32 bp) [36], which functions in *S. cerevisiae* and *Y. lipolytica*, was functionally verified in *C. tropicalis* by the GFP reporter system (Additional file 2: Fig. S4). Then the strong promoters *P*_*GAP1*_ and *P*_*FBA1*_, and terminators *T*_*synth7*_, *T*_*ENO1*_, *T*_*PGK1*_ and *T*_*ADH2*_, were used to control gene expression. To further enhance α-humulene production, genes encoding the entire α-humulene synthesis pathway (*ERG10*, *ERG13*, *tHMRG*, *ERG12*, *ERG8*, *ERG19*, *IDI1*, *ERG20* and *ZSS1*) were constitutively overexpressed in cytoplasmic and peroxisome fractions of CU-208. The α-humulene titre of the peroxisome engineered strain DP-H01 (expressing double copies of the nine genes) was 2.42 mg/L, 43.33-fold higher than HP02, and 1.70-fold higher than the strain expressing one copy of each of the nine genes in peroxisomes (Fig. 3a). These results indicate that the peroxisome-targeted α-humulene biosynthetic pathway could enhance production in *C. tropicalis*. Similarly, previous studies reported that targeting biosynthetic pathways to peroxisomes can enhance productivity and inhibit by-product formation [32, 37]. However, the α-humulene titre in the engineered *C. tropicalis* strain was much lower than that reported for *S. cerevisiae* [15]. Interestingly, strains overexpressing the entire α-humulene synthesis pathway in the cytoplasm exhibited a remarkable increase in α-humulene production. In strain DC-H01 expressing double copies of the α-humulene synthesis pathway genes in the cytoplasm, α-humulene production was improved more than fivefold compared with DP-H01, reaching 12.89 mg/L (Fig. 3a). A similar result was obtained for strain SC-H01 expressing only one copy of each α-humulene synthesis pathway gene in the cytoplasm (Fig. 3a). In addition, the cell growth of the engineered strains was significantly inhibited compared with the initial strain (Fig. 1b, a)

The peroxisome subcellular organelle is nonessential for yeast growth, and a series of studies have focused on peroxisome engineering of yeast for terpene production [15–17]. However, our current results showed that the peroxisome of *C. tropicalis* is not an ideal subcellular location for α-humulene production. Therefore, strain DC-H01 was chosen for further genetic modification to improve α-humulene biosynthesis.

### Identifying rate-limiting steps in the α-humulene pathway

Many researchers have demonstrated that cytoplasmic-engineered *S. cerevisiae* can be used for terpenoid production with high efficiency [38, 39]. Moreover, β-carotene production in cytoplasmic-engineered *Y. lipolytica* reached 6.5 g/L [40]. Our current results showed that the α-humulene titre of DC-H01 was significantly higher than that of SC-H01, indicating a bottleneck in the α-humulene biosynthesis pathway of the SC-H01 strain. However, the α-humulene titre was significantly lower (12.89 mg/L, Fig. 3a). Therefore, we hypothesised that α-humulene biosynthesis in DC-H01 may be limited by one or several steps in the pathway.

To confirm this hypothesis, three gene expression cassettes (cassette 1 for *ERG10*, *ERG13* and *tHMGR* expression; cassette 2 for *ERG12*, *ERG8*, *ERG19* and *IDI1* expression; cassette 3 for *ERG20* and *ZSS1* expression) were constructed and transformed into strain DC-H02 (a *URA3* pop-out derivative of DC-H01), generating strain DC-H03S (expressing one copy of cassette 1), DC-H03D (expressing double copies of cassette 1), DC-H05S (expressing one copy of cassette 2), DC-H05D (expressing double copies of cassette 2), DC-H07S (expressing one copy of cassette 3) and DC-H07D (expressing double copies of cassette 3). Engineered strain DC-H07S produced nearly 50% more α-humulene compared than DC-H01 (19.33 mg/L vs 12.90 mg/L; Fig. 3b), whereas strain DC-H07D produced 32.68 mg/L of α-humulene, indicating that overexpression of *ERG20* and *ZSS1* enhanced α-humulene production. Further studies indicated that the increase in α-humulene titre was mainly due to expression of *ZSS1* (Fig. 3b, strain DC-H09D overexpressing *ZSS1* vs DC-H07D). However, the effects of co-expression of *ERG10*, *ERG13* and *tHMGR*, and *ERG12*, *ERG8*, *ERG19* and *IDI1* were limited (DC-H03S, DC-H03D, DC-H05S and DC-H05D vs. DC-H01), indicating that the steps catalysed by these enzymes are not the bottlenecks for α-humulene production in strain DC-H01. Compared with DC-H01, expression of cassette 2 inhibited cell growth of strains DC-H05S and DC-H05D. It was previously reported that IPP and DMAPP are toxic to mitochondria, and higher levels of these pyrophosphorylated intermediates can inhibit the growth of cells [33]. Overexpression of *ERG12*, *ERG8*, *ERG19* and *IDI1* genes can lead to accumulation of IPP and DMAPP in DC-H05S and DC-H05D, and they may be transported from the cytoplasm to the mitochondria [41], where they disrupt mitochondrial function and inhibit cell growth.

Considering that increasing the expression of the *ZSS1* gene can significantly increase the production of α-humulene (DC-H09D vs DC-H01 and DC-H07D vs DC-H01), we speculated that increasing the copy number of the *ZSS1* gene may further improve the yield of α-humulene. Since the *GAP1* promoter is one of the strongest promoters (more than twofold stronger than the *FBA1* promoter) [25], we chose this promoter to overexpress the *ZSS1* gene. The *ZSS1* expression cassette was integrated at the D-lactate dehydrogenase gene (*DLD1b*) and/or the lipid phosphate phosphatase gene (*LPP2*) locus of strain DC-H08 (a *URA3* pop-out derivative of DC-H07D) to increase the copy number of *ZSS1*, resulting in strains DC-H11S, DC-H11D, DC-H13S and DC-H13D. As shown in Fig. 3c, compared with DC-H07, the titre of α-humulene was significantly improved with increasing *ZSS1* copy number. The maximum α-humulene levels in DC-H11S, DC-H11D, DC-H13S and DC-H13D reached 55.25, 77.00, 92.87 and 99.62 mg/L, respectively (Fig. 3c). Moreover, the engineered strains showed a slight increase in cell growth compared with DC-H07.

Lipid phosphate phosphatase is one of the main contributors to phosphate phosphatase activity in yeast. Deleting the *LPP1* gene can increase sesquiterpene levels in *S. cerevisiae* [39, 42]. However, our results showed that deleting the *LPP2* gene did not increase the α-humulene titre in *C. tropicalis* (Fig. 3c, DC-H15D vs DC-H07D). Compared with DC-H07D, the farnesol titre of strain DC-H15D was not changed significantly (data not shown). Indeed, in addition to the *LPP2* alleles, there are other phosphate phosphatases (at least three isozymes of diacylglycerol pyrophosphate phosphatase) in *C. tropicalis*.

Previous studies have shown that HMGR is the first rate-limiting enzyme in the MVA pathway, and NADH-dependent HMG-CoA reductase (NADH-HMGR) from *S. pomeroyi* has better performance for the production of sesquiterpenoid in yeast [38, 43]. To further investigate the rate-limiting step of DC-H13D for α-humulene synthesis, *tHMGR* and *NADH-HMGR* from *S. pomeroyi* were overexpressed. Compared with *tHMGR*, *NADH-HMGR* achieved a more significant increase in α-humulene titre (Fig. 3d, DC-H17D vs DC-H13D and DC-H19D vs. DC-H13D). When both *NADH-HMGR* and *ERG10* genes were overexpressed in DC-H13D (generating strain DC-H21D), an α-humulene titre of 119.07 mg/L was achieved, ~19.5% higher than that of DC-H13D (Fig. 3d).

### Fed-batch fermentation for α-humulene production

**Fig. 4:**
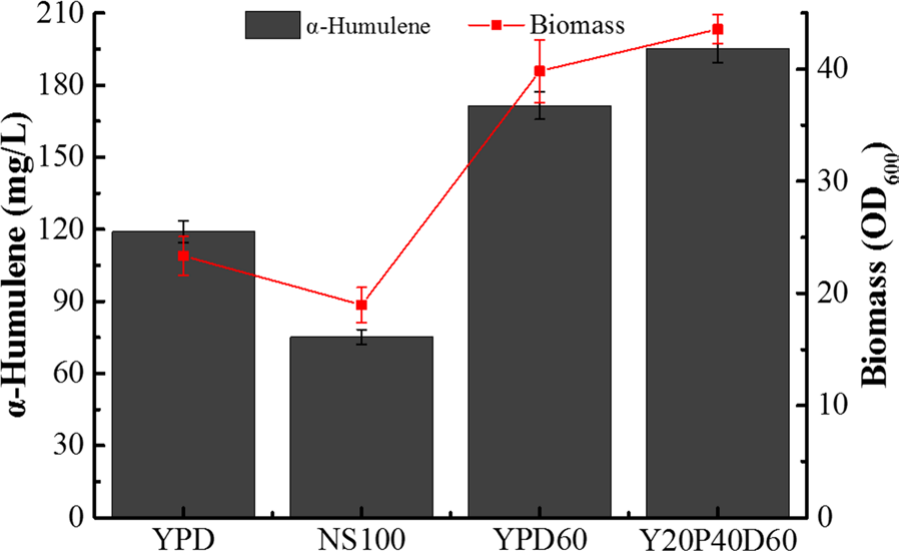
α-Humulene production in strain DC-H21D cultivated in different media. NS100 is nitrogen stress (C:N 150:1) medium described previously [23]. YPD60 is a modified YPD medium. Results are means ± standard deviations of biological triplicates

In order to improve α-humulene production of strain DC-H21D, three different types of medium, nitrogen stress medium [23] with 100 g/L glucose, YPD60 medium and Y20P40D60 medium, were tested in shake flasks prior to fed-batch fermentation. The α-humulene titre of DC-H21D was increased to 171.50 mg/L and 195.31 mg/L in YPD60 and Y20P40D60 medium, an increase of 44.0% and 64.0% compared with YPD medium (Fig. 4). Moreover, biomass was also improved. However, cell growth and α-humulene production in nitrogen stress medium were significantly lower than in YPD medium (Fig. 4).

**Fig. 5:**
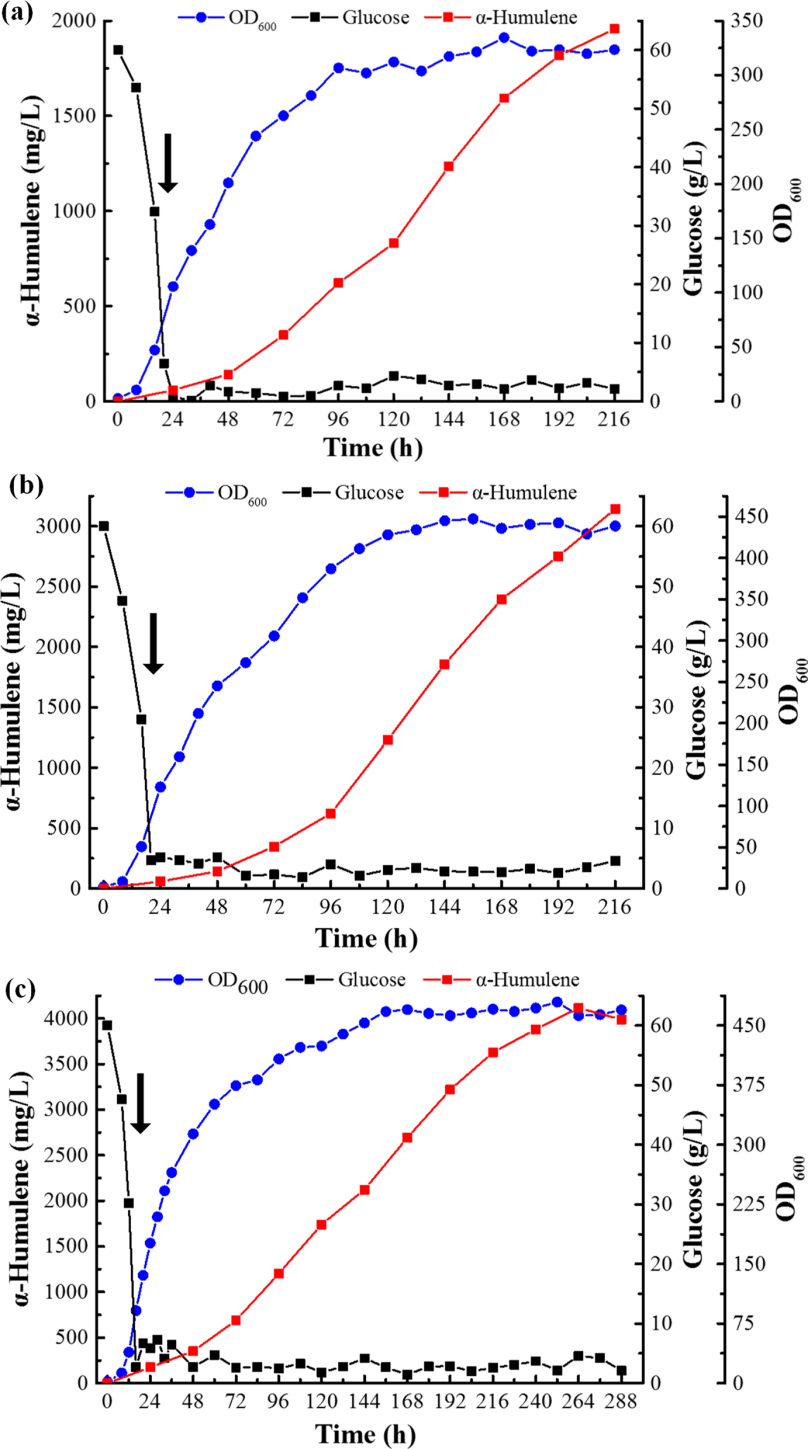
Production of α-humulene through fed-batch fermentation by strain DC-H21D in bioreactor. α-Humulene production in a 5-L bioreactor with YPD60 medium (**a)** and Y20P40D60 medium (**b)**. **c** α-Humulene production in a 30-L bioreactor with Y20P40D60 medium. The black arrow indicates the start of glucose concentration control

To further characterise α-humulene production in *C. tropicalis*, strain DC-H21D was employed for fed-batch fermentation in a 5-L bioreactor (Bailun Co., Shanghai, China) with 2 L YPD60 or Y20P40D60 medium. As shown in Fig. 5, the strain DC-H21D grew continuously in both fermentations. Finally, the maximum titre of α-humulene reached 1957.28 mg/L and 3144.37 mg/L from the YPD60 and Y20P40D60 medium, respectively, at 216 h (Fig. 5a, b). In order to further increase the titre of α-humulene, scale-up experiment was performed in a 30-L bioreactor (INFORS, Switzerland) with 12 L Y20P40D60 medium (Fig. 5c). In this fed-batch culture, glucose was quickly consumed within 16 h, and feeding was initiated at ~ 16 h after fermentation. The biomass (OD_600_) of DC-H21D increased gradually until 156 h, then fluctuated between 460 and 480 until the end of the fermentation. The concentration of α-humulene steadily increased throughout the cultivation period, and a maximum titre of 4115.42 mg/L was achieved in 264 h of fermentation. These results demonstrate the enormous potential of *C. tropicalis* to produce α-humulene and other terpenoids.

## Conclusions

In this work, the oleaginous yeast *C. tropicalis* was successfully engineered for de novo synthesis of α-humulene from glucose. Specifically, the entire α-humulene synthesis pathway was overexpressed in the cytoplasm, and the rate-limiting steps of α-humulene production were identified and relieved. And our results reveal that ZSS1 was the most crucial bottleneck enzyme of α-humulene synthesis, followed by NADH-HMGR and ERG10. The final titre of α-humulene was 195.31 mg/L and 4115.42 mg/L in shake flasks and fed-batch fermentation, respectively. This study is the first to report on terpenoid synthesis by systematic metabolic engineering of *C. tropicalis*, and the α-humulene titre achieved is highest reported to date. The findings present a platform for the industrial biosynthesis of α-humulene and other terpenoids.

## Materials and methods

### Strains, media and culture conditions

The uracil auxotrophic *C. tropicalis* CU-206 strain derived from *C. tropicalis* ATCC20336 was used as the parent strain for metabolic engineering [25]. The medium (MM, SM, FOA-SM, 2 × YPD) and culture conditions used for genetic manipulation of *C. tropicalis* were as described previously [25]. YPD60 (10 g/L yeast extract, 20 g/L peptone, 60 g/L glucose, 0.5 g/L MgSO_4_), Y20P40D60 (20 g/L yeast extract, 40 g/L peptone, 60 g/L glucose, 0.5 g/L MgSO_4_) and nitrogen stress medium (NS100, 0.5 g/L yeast extract, 0.4 g/L MgSO_4_·7H_2_O, 0.5 g/L CaCl_2_, 2 g/L KH_2_PO_4_, 0.05 g/L CuSO_4_·5H_2_O, 0.5 g/L (NH_4_)_2_SO_4_, 100 g/L glucose) medium were used for fermentation.

*E. coli* JM109 served used as the host for plasmid construction.

### Construction of plasmids and strains

The *Zingiber zerumbet* Smith α-humulene synthase gene (*ZSS1*; GenBank: AB247331.1) and the *Silicibacter pomeroyi* NADH-dependent HMG-CoA reductase gene (*NADH-HMGR*; NCBI Reference Sequence: WP_011241944.1) were codon-optimised and synthesised by Genewiz (Suzhou, China). The promoters *(P*_*GAP1*_ and *P*_*FBA1*_), terminators (*T*_*ENO1*_, *T*_*PGK1*_ and *T*_*ADH2*_) [25] and coding sequences of the eight genes involved in the FPP synthesis pathway (*ERG10*, encoding acetoacetyl-CoA thiolase; *ERG13*, encoding hydroxymethylglutaryl-CoA synthase; *HMRG*, encoding hydroxymethylglutaryl-CoA reductase; *ERG12*, encoding mevalonate kinase; *ERG8*, encoding phosphomevalonate kinase; *ERG19*, encoding mevalonate diphosphate decarboxylase; *IDI1*, encoding isopentenyl diphosphate isomerase; and *ERG20*, encoding geranyl/farnesyl diphosphate synthase) were amplified from *C. tropicalis* ATCC20336. The short synthetic terminator *T*_*synth7*_ [36] was created by adding the sequence to primers. Previous studies found that knockout of *POX5* (encoding acyl-CoA oxidase), *ALD1* (encoding fatty aldehyde dehydrogenase), *FAO1* (encoding alcohol oxidase), *DLD1a* and *DLD1b* (encoding D-lactate dehydrogenase), and *LPP2* (encoding diacylglycerol pyrophosphate phosphatase 2) have little effect on cells growth when cultured with glucose as the sole carbon source [44, 45], therefore we selected these loci for genomic integration of the α-humulene synthesis pathway. Single guide RNA expression cassettes targeting these genome sites were constructed using the method described in our previous work [25]. Plasmids were constructed according to standard restriction enzyme-based cloning or using a ClonExpress II One Step Cloning Kit (Vazyme, Nanjing, China). The detailed process for plasmid construction is described in the Additional file 1. All primers used in this study are listed in Additional file 3: Table S1. To obtain a higher strain construction efficiency, the codon-optimised *Cas9* expression cassette CAT2-gda324-URA3-P_GAP1_-CtCas9-T_ENO1_-CAT2 was first inserted into *C. tropicalis* CU-206 and integrated at the *CAT* locus via homologous recombination, resulting in strain CU-207, and the marker gene *URA3* pop-out derivative strain was named CU-208. Detailed procedures for integrating gene expression cassettes were performed according to our previous work [25]. All *C. tropicalis* strains used in this study are listed in Table 1.

**Table 1:** *C. tropicalis* strains used in this study

Strains	Genotypes	References
*C. tropicalis* ATCC 20,336	*URA3*/*URA3, CAT/CAT, FAO1/FAO, ALD1/ALD1, POX5/POX5, DLD1a/DLD1a, DLD1b/DLD1b, LPP2/LPP2, ERG9/ERG9*	ATCC
*C. tropicalis* CU-206	*C. tropicalis* ATCC 20,336, *ura3*/*ura3*	[25]
*C. tropicalis* 05-3	*C. tropicalis* ATCC 20,336, *ura3*/*ura3*, *cat::gda324/cat::gda324-URA3-P*_*GAP1*_*-yeGFP3-T*_*GAP1*_	[31]
*C. tropicalis* 01	*C. tropicalis CU-206, CAT/cat::gda-P*_*GAP1*_*-ZSS1-yeGFP3-PTS1-T*_*GAP1*_, *ALD1/ald1::gda-URA3-P*_*GAP1*_*-PEX3-mScarlet-T*_*GAP1*_	This study
*C. tropicalis* 02	*C. tropicalis CU-206, CAT/cat::gda-URA3-P*_*GAP1*_*-yeGFP3-T*_*synth7*_	This study
CU-207	*C. tropicalis CU-206, CAT/cat::gda-URA3-P*_*GAP1*_*-Cas9-3* × *SV40-T*_*ENO1*_	This study
CU-208	*CU-207, CAT/cat::gda-P*_*GAP1*_*-Cas9-3* × *SV40-T*_*ENO1*_	This study
HC01	CU-208, *POX5/pox5::gda-URA3-P*_*FBA1*_*-ZSS1-T*_*ADH2*_	This study
HC02	CU-208, *pox5::gda-URA3-P*_*FBA1*_*-ZSS1-T*_*ADH2*_*/pox5::gda-URA3-P*_*FBA1*_*-ZSS1-T*_*ADH2*_	This study
HC03	CU-208, *pox5::gda-P*_*FBA1*_*-ZSS1-T*_*ADH2*_*/pox5::gda-P*_*FBA1*_*-ZSS1-T*_*ADH2*_	This study
HP01	CU-208, *POX5/pox5::gda-URA3-P*_*FBA1*_*-ZSS1-PTS1-T*_*ADH2*_	This study
HP02	CU-208, *pox5::gda-URA3-P*_*FBA1*_*-ZSS1-PTS1-T*_*ADH2*_*/pox5::gda-URA3-P*_*FBA1*_*-ZSS1-PTS1-T*_*ADH2*_	This study
HC04	HC03, *fao1::gda-URA3-P*_*GAP1*_*-ERG10-T*_*ENO1*_*/fao1::gda-URA3-P*_*GAP1*_*-ERG10-T*_*ENO1*_	This study
HC05	HC03, *fao1::gda-URA3-P*_*GAP1*_*-HMGR-T*_*synth7*_*/fao1::gda-URA3-P*_*GAP1*_*-HMGR-T*_*synth7*_	This study
HC06	HC03, *fao1::gda-URA3-P*_*GAP1*_*-tHMGR-T*_*synth7*_*/fao1::gda-URA3-P*_*GAP1*_*-tHMGR-T*_*synth7*_	This study
HC07	HC03, *ERG9/erg9::gda-URA3*	This study
SC-H01	CU-208, *FAO1/fao1::gda-T*_*PGK1*_*-ERG13-P*_*FBA1*_*-P*_*GAP1*_*-tHMGR-T*_*synth7*_*-P*_*GAP1*_*-ERG10-T*_*ENO1*_, *ALD1/ald1::gda-T*_*PGK1*_*-ERG12-P*_*FBA1*_*-P*_*GAP1*_*-ERG8-T*_*synth7*_*-T*_*ADH2*_*-ERG19-P*_*FBA1*_*-P*_*GAP1*_*-IDI1-T*_*ENO1*_, *POX5/pox5::gda-URA3-P*_*GAP1*_*-ERG20-T*_*synth7*_*-T*_*ADH2*_*-ZSS1-P*_*FBA1*_	This study
DC-H01	CU-208, *fao1::gda-T*_*PGK1*_*-ERG13-P*_*FBA1*_*-P*_*GAP1*_*-tHMGR-T*_*synth7*_*-P*_*GAP1*_*-ERG10-T*_*ENO1*_*/fao1::gda-T*_*PGK1*_*-ERG13-P*_*FBA1*_*-P*_*GAP1*_*-tHMGR-T*_*synth7*_*-P*_*GAP1*_*-ERG10-T*_*ENO1*_, *ald1::gda-T*_*PGK1*_*-ERG12-P*_*FBA1*_*-P*_*GAP1*_*-ERG8-T*_*synth7*_*-T*_*ADH2*_*-ERG19-P*_*FBA1*_*-P*_*GAP1*_*-IDI1-T*_*ENO1*_*/ald1::gda-T*_*PGK1*_*-ERG12-P*_*FBA1*_*-P*_*GAP1*_*-ERG8-T*_*synth7*_*-T*_*ADH2*_*-ERG19-P*_*FBA1*_*-P*_*GAP1*_*-IDI1-T*_*ENO1*_, *pox5::gda-URA3-P*_*GAP1*_*-ERG20-T*_*synth7*_*-T*_*ADH2*_*-ZSS1-P*_*FBA1*_*/pox5::gda-URA3-P*_*GAP1*_*-ERG20-T*_*synth7*_*-T*_*ADH2*_*-ZSS1-P*_*FBA1*_	This study
DC-H02	CU-208, *fao1::gda-T*_*PGK1*_*-ERG13-P*_*FBA1*_*-P*_*GAP1*_*-tHMGR-T*_*synth7*_*-P*_*GAP1*_*-ERG10-T*_*ENO1*_*/fao1::gda-T*_*PGK1*_*-ERG13-P*_*FBA1*_*-P*_*GAP1*_*-tHMGR-T*_*synth7*_*-P*_*GAP1*_*-ERG10-T*_*ENO1*_, *ald1::gda-T*_*PGK1*_*-ERG12-P*_*FBA1*_*-P*_*GAP1*_*-ERG8-T*_*synth7*_*-T*_*ADH2*_*-ERG19-P*_*FBA1*_*-P*_*GAP1*_*-IDI1-T*_*ENO1*_*/ald1::gda-T*_*PGK1*_*-ERG12-P*_*FBA1*_*-P*_*GAP1*_*-ERG8-T*_*synth7*_*-T*_*ADH2*_*-ERG19-P*_*FBA1*_*-P*_*GAP1*_*-IDI1-T*_*ENO1*_, *pox5::gda-P*_*GAP1*_*-ERG20-T*_*synth7*_*-T*_*ADH2*_*-ZSS1-P*_*FBA1*_*/pox5::gda-P*_*GAP1*_*-ERG20-T*_*synth7*_*-T*_*ADH2*_*-ZSS1-P*_*FBA1*_	This study
SP-H01	CU-208, *FAO1/fao1::gda-T*_*PGK1*_*-PTS1-ERG13-P*_*FBA1*_*-P*_*GAP1*_*-tHMGR-PTS1-T*_*synth7*_*-P*_*GAP1*_*-ERG10-PTS1-T*_*ENO1*_, *ALD1/ald1::gda-T*_*PGK1*_*-PTS1-ERG12-P*_*FBA1*_*-P*_*GAP1*_*-ERG8-PTS1-T*_*synth7*_*-T*_*ADH2*_*-PTS1-ERG19-P*_*FBA1*_*-P*_*GAP1*_*-IDI1-PTS1-T*_*ENO1*_, *POX5/pox5::gda-URA3-P*_*GAP1*_*-ERG20-PTS1-T*_*synth7*_*-T*_*ADH2*_*-PTS1-ZSS1-P*_*FBA1*_	This study
DP-H01	CU-208, *fao1::gda-T*_*PGK1*_*-PTS1-ERG13-P*_*FBA1*_*-P*_*GAP1*_*-tHMGR-PTS1-T*_*synth7*_*-P*_*GAP1*_*-ERG10-PTS1-T*_*ENO1*_*/fao1::gda-T*_*PGK1*_*-PTS1-ERG13-P*_*FBA1*_*-P*_*GAP1*_*-tHMGR-PTS1-T*_*synth7*_*-P*_*GAP1*_*-ERG10-PTS1-T*_*ENO1*_, *ald1::gda-T*_*PGK1*_*-PTS1-ERG12-P*_*FBA1*_*-P*_*GAP1*_*-ERG8-PTS1-T*_*synth7*_*-T*_*ADH2*_*-PTS1-ERG19-P*_*FBA1*_*-P*_*GAP1*_*-IDI1-PTS1-T*_*ENO1*_*/ald1::gda-T*_*PGK1*_*-PTS1-ERG12-P*_*FBA1*_*-P*_*GAP1*_*-ERG8-PTS1-T*_*synth7*_*-T*_*ADH2*_*-PTS1-ERG19-P*_*FBA1*_*-P*_*GAP1*_*-IDI1-PTS1-T*_*ENO1*_, *pox5::gda-URA3-P*_*GAP1*_*-ERG20-PTS1-T*_*synth7*_*-T*_*ADH2*_*-PTS1-ZSS1-P*_*FBA1*_*/pox5::gda-URA3-P*_*GAP1*_*-ERG20-PTS1-T*_*synth7*_*-T*_*ADH2*_*-PTS1-ZSS1-P*_*FBA1*_	This study
DC-H03S	DC-H02, *DLD1a/dld1a::gda-URA3-T*_*PGK1*_*-ERG13-P*_*FBA1*_*-P*_*GAP1*_*-tHMGR-T*_*synth7*_*-P*_*GAP1*_*-ERG10-T*_*ENO1*_	This study
DC-H03D	DC-H02, *dld1a::gda-URA3-T*_*PGK1*_*-ERG13-P*_*FBA1*_*-P*_*GAP1*_*-tHMGR-T*_*synth7*_*-P*_*GAP1*_*-ERG10-T*_*ENO1*_*/dld1a::gda-URA3-T*_*PGK1*_*-ERG13-P*_*FBA1*_*-P*_*GAP1*_*-tHMGR-T*_*synth7*_*-P*_*GAP1*_*-ERG10-T*_*ENO1*_	This study
DC-H05S	DC-H02, *DLD1a/dld1a::gda-URA3-T*_*PGK1*_*-ERG12-P*_*FBA1*_*-P*_*GAP1*_*-ERG8-T*_*synth7*_*-T*_*ADH2*_*-ERG19-P*_*FBA1*_*-P*_*GAP1*_*-IDI1-T*_*ENO1*_	This study
DC-H05D	DC-H02, *dld1a::gda-URA3-T*_*PGK1*_*-ERG12-P*_*FBA1*_*-P*_*GAP1*_*-ERG8-T*_*synth7*_*-T*_*ADH2*_*-ERG19-P*_*FBA1*_*-P*_*GAP1*_*-IDI1-T*_*ENO1*_*/dld1a::gda-URA3-T*_*PGK1*_*-ERG12-P*_*FBA1*_*-P*_*GAP1*_*-ERG8-T*_*synth7*_*-T*_*ADH2*_*-ERG19-P*_*FBA1*_*-P*_*GAP1*_*-IDI1-T*_*ENO1*_	This study
DC-H07S	DC-H02, *DLD1a/dld1a::gda-URA3-P*_*GAP1*_*-ERG20-T*_*synth7*_*-T*_*ADH2*_*-ZSS1-P*_*FBA1*_	This study
DC-H07D	DC-H02, *dld1a::gda-URA3-P*_*GAP1*_*-ERG20-T*_*synth7*_*-T*_*ADH2*_*-ZSS1-P*_*FBA1*_*/dld1a::gda-URA3-P*_*GAP1*_*-ERG20-T*_*synth7*_*-T*_*ADH2*_*-ZSS1-P*_*FBA1*_	This study
DC-H08	DC-H02, *dld1a::gda-P*_*GAP1*_*-ERG20-T*_*synth7*_*-T*_*ADH2*_*-ZSS1-P*_*FBA1*_*/dld1a::gda-P*_*GAP1*_*-ERG20-T*_*synth7*_*-T*_*ADH2*_*-ZSS1-P*_*FBA1*_	This study
DC-H09D	DC-H02, *dld1a::gda-URA3-T*_*ADH2*_*-ZSS1-P*_*FBA1*_*/dld1a::gda-URA3-T*_*ADH2*_*-ZSS1-P*_*FBA1*_	this study
DC-H11S	DC-H08, *LPP2/lpp2::gda-URA3-P*_*GAP1*_*-ZSS1-T*_*synth7*_	This study
DC-H11D	DC-H08, *lpp2::gda-URA3-P*_*GAP1*_*-ZSS1-T*_*synth7*_*/lpp2::gda-URA3-P*_*GAP1*_*-ZSS1-T*_*synth7*_	This study
DC-H12	DC-H08, *lpp2::gda-P*_*GAP1*_*-ZSS1-T*_*synth7*_*/lpp2::gda-P*_*GAP1*_*-ZSS1-T*_*synth7*_	This study
DC-H13S	DC-H12, *DLD1b/dld1b::gda-URA3-P*_*GAP1*_*-ZSS1-T*_*synth7*_	This study
DC-H13D	DC-H12, *dld1b::gda-URA3-P*_*GAP1*_*-ZSS1-T*_*synth7*_*/dld1b::gda-URA3-P*_*GAP1*_*-ZSS1-T*_*synth7*_	This study
DC-H14	DC-H12, *dld1b::gda-P*_*GAP1*_*-ZSS1-T*_*synth7*_*/dld1b::gda-P*_*GAP1*_*-ZSS1-T*_*synth7*_	This study
DC-H15D	DC-H08, *lpp2::gda-URA3/lpp2::gda-URA3*	This study
DC-H17D	DC-H12, *dld1b::gda-URA3-P*_*FBA1*_*-tHMGR-T*_*ADH2*_*-T*_*synth7*_*-ZSS1-P*_*GAP1*_*/dld1b::gda-URA3-P*_*FBA1*_*-tHMGR-T*_*ADH2*_*-T*_*synth7*_*-ZSS1-P*_*GAP1*_	This study
DC-H19D	DC-H12, *dld1b::gda-URA3-P*_*FBA1*_*-NADH-HMGR-T*_*ADH2*_*-T*_*synth7*_*-ZSS1-P*_*GAP1*_*/dld1b::gda-URA3-P*_*FBA1*_*-NADH-HMGR-T*_*ADH2*_*-T*_*synth7*_*-ZSS1-P*_*GAP1*_	This study
DC-H21D	DC-H12, *dld1b::gda-URA3-T*_*PGK1*_*-NADH-HMGR-P*_*FBA1*_*-P*_*GAP1*_*-ZSS1-T*_*synth7*_*-P*_*GAP1*_*-ERG10-T*_*ENO1*_*/ dld1b::gda-URA3-T*_*PGK1*_*-NADH-HMGR-P*_*FBA1*_*-P*_*GAP1*_*-ZSS1-T*_*synth7*_*-P*_*GAP1*_*-ERG10-T*_*ENO1*_	This study

### α-Humulene fermentation in shake flasks

To produce α-humulene, yeast cells were pre-cultured in 100-mL shake flasks containing 20 mL YPD medium with shaking at 200 rpm and 30 °C (until the OD_600_ value reached 10–15). Logarithmic phase cultures were diluted to an initial OD_600_ of 0.1 in 30 mL YPD medium and cultivated at 200 rpm and 30 °C. Next, 10% (v/v) *n*-dodecane was added to the culture aseptically after 10 h. After 96 h of fermentation, the liquid culture was centrifuged at 5000 g for 5 min. The upper organic layer was collected for volume measurement and α-humulene quantification. All experiments were performed in triplicate.

### Fed-batch fermentation of α-humulene

Fed-batch fermentation was performed in a 30-L bioreactor (INFORS, Switzerland). Firstly, a single colony of *C. tropicalis* DC-H21D was pre-cultured in a 250-mL shake flask containing 50 mL YPD medium for 20 h. The resulting culture was diluted in 100 mL of fresh YPD medium to an initial OD_600_ of 0.5 in a 500 mL shake flask and cultivated at 200 rpm and 30 °C for 12 h. The entire 1000 mL seed culture was inoculated into a 30-L bioreactor containing 12 L of Y20P40D60 medium. Fermentation for α-humulene production was performed at 30 °C and the pH was maintained at 5.5 using ammonium hydroxide. Dissolved oxygen was maintained above 20% by adjusting the agitation (300–900 rpm) and the air flow rate (2–4 vvm). When the glucose concentration dipped below 5 g/L for the first time, concentrated glucose solution (80%, w/v) was added to the yeast culture to provide adequate carbon source (below 5 g/L). For two-phase extractive fermentation, 10% (v/v) *n*-dodecane was added to the culture after 12 h of fermentation.

### Analytical methods

Glucose was quantified using a biosensor (Biology Institute of Shandong Academy of Sciences, Shandong, China). The biomass (OD_600_) of *C. tropicalis* was determined using a UV-2000 spectrophotometer (UNIC, Shanghai, China).

For qualitative analysis of α-humulene in fed-batch fermentation culture, 5 mL of fermentation broth was centrifuged at 5000 g for 5 min, and cell pellets were resuspended in ddH_2_O and lysed with an ultrasonic cell disruptor. The culture supernatant and cell lysate were extracted with 10 mL of *n-*hexane for 5 min at room temperature with agitation. The *n-*hexane extract was appropriately diluted with *n-*hexane and residual water was removed with Na_2_SO_4_. α-Humulene was quantified with a Trace1310 Triple Quadrupole GC–MS system equipped with a gas chromatograph (Thermo Scientific, Waltham, MA, USA), and a TG-5 ms column (Thermo Scientific) connected to a TSQ8000 mass spectrometer (Thermo Scientific). Measurements were conducted with a helium flow maintained at 1.2 mL/min, split injections (10:1) at 280 °C, an initial column temperature of 50 °C maintained for 1 min, then increased to 180 °C at a rate of 10 °C/min, then increased to 280 °C at 25 °C/min and maintained for 6 min.

## Supplementary Information

**Additional file 1:** Additional methods for plasmid construction.

**Additional file 2:** **Fig S1. **GC-MS analysis of α-humulene from the dodecane of the cultures in engineered *C. tropicalis*. **Fig S2 **Bioinformatic analysis of *HMGR1* protein from *C. tropicalis* ATCC20336. **Fig S3 **Transcription levels of the *ERG9* gene in HC02 and HC07 strains. **Fig S4 **Confirmation of the synthetic terminator *T*_*synth7*_ using GFP as a reporter.

**Additional file 3:** **Table S1.** Primers used for plasmids construction.

## Data Availability

All data generated or analysed during this study are included in this published article and its Additional information files.

## Abbreviations

DCW: Dry cell weight
ZSS1: α-Humulene synthase
MVA: Mevalonate
MEP: Methylerythritol phosphate
carB: Phytoene dehydrogenase
carRP: Phytoene synthase and lycopene cyclase
FPP: Farnesyl diphosphate
CRISPR–Cas9: Clustered regularly interspaced short palindromic repeat (CRISPR) and CRISPR-associated protein 9
CRISPRi: CRISPR interference
GC–MS: Gas chromatography–mass spectrometry
POX5: Acyl-CoA oxidase
ALD1: Fatty aldehyde dehydrogenase
FAO1: Alcohol oxidase
CAT: Carnitine acetyltransferase;
DLD1a: d-Lactate dehydrogenase 1a
DLD1b: d-Lactate dehydrogenase 1b
LPP1: Lipid phosphate phosphatase 1
LPP2: Lipid phosphate phosphatase 2
*P*_*GAP1*_: Promoter of glyceraldehyde-3-phosphate dehydrogenase gene
*P*_*FBA1*_: Promoter of fructose bisphosphate aldolase gene
*T*_*synth7*_: Short synthetic terminator
*T*_*ENO1*_: Terminator of enolase gene
*T*_*PGK1*_: Terminator of phosphoglycerate kinase gene
*T*_*ADH2*_: Terminator of alcohol dehydrogenase gene
GFP: Green fluorescent protein
PTS1: Peroxisome targeting signal-1
HMGR: Hydroxymethylglutaryl-CoA reductases
tHMGR: n-terminal truncated HMGR
NADH-HMGR: NADH-dependent HMGR
ERG10: Acetoacetyl-CoA thiolase
ERG9: Squalene synthase
ERG13: Hydroxymethylglutaryl-CoA synthase
ERG12: Mevalonate kinase
ERG8: Phosphomevalonate kinase
ERG19: Mevalonate diphosphate decarboxylase
IDI1: Isopentenyl diphosphate isomerase
ERG20: Geranyl/farnesyl diphosphate synthase
URA3: Orotidine monophosphate decarboxylase
RT-qPCR: Real-time quantitative PCR
