# Supplementary material for: Engineering the oleaginous yeast Candida tropicalis for α-humulene overproduction
Source: Biotechnol Biofuels Bioprod. 2022 May 26;15:59. doi: 10.1186/s13068-022-02160-8 (PMC9137083; doi:10.1186/s13068-022-02160-8)
Supplement: Supplementary file 1 — Additional file 1: Additional methods for plasmid construction. [file 13068_2022_2160_MOESM1_ESM.docx]

**Engineering the** **oleaginous yeast** ***Candida tropicalis* for α-humulene overproduction**

Lihua Zhang^1^, Haiquan Yang^1^, Yuanyuan Xia^1^, Wei Shen^1^, Liming Liu^1^, Qi Li^1*^, Xianzhong Chen^1*^

^1^Key Laboratory of Industrial Biotechnology, Ministry of Education, & School of Biotechnology, Jiangnan University, Wuxi 214122, People’s Republic of China

*Correspondence author:

Key Laboratory of Industrial Biotechnology, Ministry of Education, Jiangnan University, Wuxi 214122, People’s Republic of China.

*E-mail* address: [liqi@jiangnan.edu.cn](mailto:liqi@jiangnan.edu.cn) (Q. Li), xzchen@jiangnan.edu.cn (X.Z. Chen)

Tel: +86-510-85918122

Fax: +86-510-85918122

Address: School of Biotechnology, Jiangnan University, 1800 Lihu Road, Wuxi 214122, People’s Republic of China

**Additional methods for plasmid construction**

**Construction of Ts-CAT2-gda-URA3-P_GAP1_-CtCas9-T_ENO1_**

The plasmid Ts-P_GAP1_-CtCas9-T_ENO1_ [[1](#_ENREF_1)] was digested with *Spe*I to generate the P_GAP1_-CtCas9-T_ENO1_ cassette. After blunting its sticky ends using *pfu* DNA polymerase (BBI, Shanghai, China), the cassette was inserted into plasmid Ts-CAT2-gda-URA3 [[2](#_ENREF_2)] digested with *Xba*I, and had its sticky ends blunted, yielding plasmid Ts-CAT2-gda-URA3-P_GAP1_-CtCas9-T_ENO1_. This plasmid was digested with *Mlu*I to generate the CAT2-gda-URA3-P_GAP1_-CtCas9-T_ENO1_-CAT2 cassette.

**Construction of Ts-CAT2-gda-URA3-P_GAP1_-ZSS1-yeGFP3**-PTS1**-T_GAP1_**

The peroxisomal targeting signal (PTS1, amino acid sequence SKL) was chemically synthesized (anneal using primers SKL-F and SKL-R) and inserted into Ts-CAT2-gda324-URA3-P_GAP1_-yeGFP3-T_GAP1_ [[2](#_ENREF_2)] between its *yeGFP3* gene and *T_GAP1_* terminator by ClonExpress^®^ II One Step Cloning Kit (Vazyme, Nanjing, China), to generate plasmid Ts-CAT2-gda324-URA3-P_GAP1_-yeGFP3-PTS1-T_GAP1_.

The open reading frame (ORF) of the α-humulene synthase gene (*ZSS1*, GenBank accession number: AB247331.1) from *Zingiber zerumbet* was optimized using *C. tropicalis* codon usage bias and chemically synthesized (Sequence S1). And the optimized *ZSS1* gene was inserted into pMD19-T Simple (TaKaRa, Dalian, China) to generate plasmid Ts-ZSS1. The ORF of the *ZSS1* was amplified from plasmid *Ts-ZSS1* using PCR with primers ZSS-F and ZSS-R, and inserted into Ts-CAT2-gda324-URA3-P_GAP1_-yeGFP3-PTS1-T_GAP1_ between its *yeGFP3* gene and *P_GAP1_* promoter by the One Step Cloning Kit, to generate plasmid Ts-CAT2-gda324-URA3-P_GAP1_-ZSS1-yeGFP3-PTS1-T_GAP1_.

**Construction of Ts-ALD1-gda-URA3-P_GAP1_-PEX3-mScarlet-T_GAP1_**

The fatty aldehyde dehydrogenase gene (*ALD1*) was amplified from *C. tropicalis* ATCC 2033 genomic DNA using PCR using primers ALD-F1 and ALD-R1, and ligated into the plasmid vector pMD 19-T Simple to yield plasmid Ts- ALD1. Then, inverse PCR with primers rALD-F1 and rALD-R1 was used to eliminate the middle region of ALD1 (184 bp). The resulting PCR product (ALD1-Ts-ALD1) was digested with *Pst*I and *Xba*I and ligated to gda324-URA3-20-20 cassettes yielding plasmid Ts-ALD1-gda-URA3-20-20.

The ORF of the red fluorescence protein gene (*mScarlet*, GenBank accession number: MK427053.1) was chemically synthesized and inserted into Ts-P_GAP1_-yeGFP3-T_GAP1_ [[2](#_ENREF_2)] to replace its *yeGFP3* gene, to generate plasmid Ts-P_GAP1_-mScarlet-T_GAP1_. The ORF of the peroxisome membrane protein gene (*PEX3*) was amplified from the *C. tropicalis* ATCC 20336 genome using PCR with primers PEX3-F and PEX3-R, and inserted into Ts-P_GAP1_-mScarlet-T_GAP1_ between its *P_GAP1_* promoter and *mScarlet* gene by the One Step Cloning Kit, to generate plasmid Ts-P_GAP1_-PEX3-mScarlet-T_GAP1_. The P_GAP1_-PEX3-mScarlet-T_GAP1_ cassette was isolated from plasmid Ts-P_GAP1_-PEX3-mScarlet-T_GAP1_. After blunting its sticky ends using *pfu* DNA polymerase, the cassette was inserted into plasmid Ts-ALD1-gda-URA3-20-20 that had been digested with *Xba*I and *Eco*RI, and had its sticky ends blunted, yielding plasmid Ts-ALD1-gda-URA3-P_GAP1_-PEX3-mScarlet-T_GAP1_.

**Construction of Ts-CAT2-gda-URA3-P_GAP1_-yeGFP3-T_synth7_**

The short synthetic terminator *T_synth7_* (TATATAACTGTCTAGAAATAAATTTTTTCAAA) [[3](#_ENREF_3)] was chemically synthesized (anneal using primers TSYN7-F and TSYN7-R) and inserted into Ts-CAT2-gda324-URA3-P_GAP1_-yeGFP3-T_GAP1_ to replace the *T_GAP1_* terminator by ClonExpress^®^ II One Step Cloning Kit, yielding plasmid Ts-CAT2-gda324-URA3-P_GAP1_-yeGFP3-*T_synth7_*.

**Construction of PZE120-01**

The alcohol dehydrogenase gene terminator (*T_ADH2_*) was amplified from the *C. tropicalis* ATCC 20336 genome using PCR with primers T7TA2-F and T7TA2-R, and the result DNA fragment contains the *T_ADH2_* terminator and a 32 bp short synthetic terminators *T_synth7_*. Then the DNA fragment was inserted into pMD19-T Simple to generate plasmid Ts-T_ADH2_-T_synth7_.

The fructose bisphosphate aldolase gene promoter (*P_FBA1_*) was amplified from the *C. tropicalis* ATCC 20336 genome using PCR with primers PFBA-F1 and PFBA-R1, and inserted into *Not*I and *Eco*RI digested Ts-ZSS1 to generate plasmid Ts-P_FBA1_-ZSS1. This plasmid was digested with *Sac*I and *Not*I to generate the P_FBA1_-ZSS1, and inserted into similarly digested Ts-T_ADH2_-T_synth7_ to generate plasmid Ts-P_FBA1_-ZSS1-T_ADH2_-T_synth7_.

The promoter of the glyceraldehyde-3-phosphate dehydrogenase gene (*P_GAP1_*) was amplified from the *C. tropicalis* ATCC 20336 genome using PCR with primers PGAP-F and PGAP-R, and inserted into pMD19-T Simple to generate plasmid Ts-P_GAP1_. The plasmid was linearized by *Sal*I. The *ERG20* gene was amplified from the genomic DNA of *C. tropicalis* ATCC 20336 (using primer sets ERG20-F and ERG20-R), which contain 15-20 base pairs (bp) of homology with the linearized Ts-P_GAP1_ allow inserted into the linearized plasmid (by ClonExpress® II One Step Cloning Kit, Vazyme, Nanjing, China), resulting in plasmid Ts-P_GAP1_-ERG20. This plasmid was digested with *Spe*I and *Xba*I to generate the P_GAP1_-ERG20, and inserted into similarly digested Ts-P_FBA1_-ZSS1-T_ADH2_-T_synth7_ to generate plasmid Ts-P_FBA1_-ZSS1-T_ADH2_-T_synth7_-ERG20-P_GAP1_ (PZE120-01 in short).

**Construction of PZE120-03-2**

The fusion-protein gene (ZSS1-PTS1) was amplified from plasmid Ts-ZSS1 using PCR with primers ZSS1-F and ZSS1-R, and inserted into PZE120-01 at its *Eco*RI and *Sac*I sites, yielding plasmid Ts-P_FBA1_-ZSS1-PTS1-T_ADH2_-T_synth7_-ERG20-P_GAP1_ (PZE120-03-1 in short). The fusion-protein gene (ERG20-PTS1) was amplified from *C. tropicalis* ATCC 20336 genomic DNA using PCR with primers ERG20-F and ERG20-R2, and inserted into PZE120-01-3-1 at its *Kpn*I and *Spe*I sites, yielding plasmid Ts-P_FBA1_-ZSS1-PTS1-T_ADH2_-T_synth7_-PTS1-ERG20-P_GAP1_ (PZE120-03-2 in short).

**Construction of POX5-P_FBA1_-ZSS1-T_ADH2_**

Firstly, a DNA fragment containing 20 bp of homology with *T_ENO1_* terminator, 20 bp of homology with *T_PGK1_* terminator, and a unique *Eco*RI site were inserted into Tm-*gda324-URA3* [[2](#_ENREF_2)] before its *Xba*I site to generate plasmid Tm-gda324-URA3-20-20. The acyl-CoA oxidase gene (*POX5*) was amplified from *C. tropicalis* ATCC 20336 genomic DNA using PCR using primers POX5-F and POX5-R, and ligated into the plasmid vector pMD 19-T Simple to yield plasmid Ts-POX5. Then, inverse PCR with primers rPOX5-F and rPOX5-R was used to eliminate the middle region of POX5 (approximately 399 bp). The resulting PCR product (POX5-Ts-POX5) was digested with *Pst*I and *Xba*I and ligated to gda324-URA3-20-20 cassettes yielding plasmid Ts-POX5-gda-URA3-20-20.

The PFBA1-ZSS1-TADH2 cassette from plasmid Ts-P_FBA1_-ZSS1-T_ADH2_-T_synth7_ was amplified using PCR with primers SB-F and SB-R and then inserted into Ts-POX5-gda-URA3-20-20 at its *Eco*RI site by ClonExpress® II One Step Cloning Kit, yielding plasmid Ts-POX5-gda-URA3-P_FBA1_-ZSS1-T_ADH_ (POX5-P_FBA1_-ZSS1-T_ADH_ in short). This plasmid was digested with *Mlu*I to generate the POX5-gda-URA3-P_FBA1_-ZSS1-T_ADH2_-POX5 cassette.

**Construction of POX5-P_FBA1_-ZSS1-PTS1-T_ADH2_**

The P_FBA1_-ZSS1-PTS1-T_ADH2_ cassette from plasmid PZE120-03-2 was amplified using PCR with primers SB-F and SB-R and then inserted into Ts-POX5-gda-URA3-20-20 at its *Eco*RI site by ClonExpress® II One Step Cloning Kit, yielding plasmid Ts-POX5-gda-URA3-P_FBA1_-ZSS1-PTS1-T_ADH2_ (POX5-P_FBA1_-ZSS1-PTS1-T_ADH2_ in short). This plasmid was digested with *Mlu*I to generate the POX5-gda-URA3-P_FBA1_-ZSS1-PTS1-T_ADH2_-POX5 cassette.

**Construction of PHMGR-01, PtHMGR-01 and PtHMGR-03**

The ORF of the hydroxymethylglutaryl-CoA reductase (*HMGR*) was amplified from the *C. tropicalis* ATCC 20336 genome using PCR with primers HMGR-F and HMGR-R, and inserted into PZE120-01 at its *Kpn*I and *Spe*I sites, yielding plasmid Ts-P_FBA1_-ZSS1-T_ADH2_-T_synth7_-HMGR-P_GAP1_ (PHMGR-01 in short). A similar strategy was used for the construction of plasmid Ts-P_FBA1_-ZSS1-T_ADH2_-T_synth7_-tHMGR-P_GAP1_ (PtHMGR-01 in short) and Ts-P_FBA1_-ZSS1-T_ADH2_-T_synth7_-PTS1-tHMGR-P_GAP1_ (PtHMGR-03 in short), which contain the truncated HMGR (tHMGR) expression cassettes.

**Construction of FAO1-T_synth7_-HMGR-P_GAP1_ and FAO1-T_synth7_-tHMGR-P_GAP1_**

The alcohol oxidase gene (*FAO1*) was amplified from *C. tropicalis* ATCC 20336 genomic DNA using PCR using primers FAO1-F1 and FAO1-R1, and ligated into the plasmid vector pMD 19-T Simple to yield plasmid Ts-FAO1. Then, inverse PCR with primers rFAO1-F1 and rFAO1-R1 was used to eliminate the middle region of *FAO1* (44 bp). The resulting PCR product (FAO1-Ts-FAO1) was digested with *Pst*I and *Xba*I and ligated to gda324-URA3-20-20 yielding plasmid Ts-FAO1-gda-URA3-20-20.

The *ZSS1* expression cassette (T_synth7_-HMGR-P_GAP1_) was amplified from plasmid PHMGR-01 with primers St-F and St-R, and then inserted into plasmid Ts-FAO1-gda-URA3-20-20 at its *Eco*RI site by ClonExpress® II One Step Cloning Kit, yielding plasmid Ts-FAO1-gda-URA3-T_synth7_-HMGR-P_GAP1_ (FAO1-T_synth7_-HMGR-P_GAP1_ in short). A similar strategy was used for the construction of plasmid Ts-FAO1-gda-URA3-T_synth7_-tHMGR-P_GAP1_ (FAO1-T_synth7_-tHMGR-P_GAP1_ in short). The plasmid was FAO1-T_synth7_-HMGR-P_GAP1_ (or FAO1-T_synth7_-tHMGR-P_GAP1_) was digested with *Mlu*I to generate the HMGR expression cassettes FAO1-gda-URA3-T_synth7_-HMGR-P_GAP1_-FAO1 (or FAO1-gda-URA3-T_synth7_-tHMGR-P_GAP1_-FAO1).

**Construction of FAO1-P_GAP1_-ERG10-T_ENO1_**

The terminator of the phosphoglycerate kinase gene (*T_PGK1_*) was amplified from the *C. tropicalis* ATCC 20336 genome using PCR with primers TPGK1-F and TPGK1-R, and then inserted into Ts-P_FBA1_-CtcarB-T_FBA1_ [[1](#_ENREF_1)] between its *Xho*I and *Xba*I sites to generate plasmid Ts-P_FBA1_-CtcarB-T_PGK1_. This plasmid was digested with *Not*I and *Xba*I to generate the P_FBA1_-CtcarB-T_PGK1_ cassette, and inserted into similarly digested Ts-P_GAP1_-CtcarRP-T_ENO1_ [[1](#_ENREF_1)] to generate plasmid Ts-T_PGK1_-CtcarB-P_FBA1_-P_GAP1_-CtcarRP-T_ENO1_ (PBRP-01 in short).

The ORF of the acetoacetyl-CoA thiolase gene (*ERG10*) was amplified from the *C. tropicalis* ATCC 20336 genome using PCR with primers ERG10-F and ERG10-R, and inserted into *Sal*I and *Nhe*I digested PBRP-01 to generate plasmid Ts-T_PGK1_-CtcarB-P_FBA1_-P_GAP1_-ERG10-T_ENO1_ (PEE1013-01-1 in short). The *ERG10* expression cassette (P_GAP1_-ERG10-T_ENO1_) was amplified from plasmid PEE1013-01-1 with primers St-F and TENO1-R, then inserted into plasmid Ts-FAO1-gda-URA3-20-20 at its *Eco*RI site by ClonExpress® II One Step Cloning Kit, yielding plasmid Ts-FAO1-gda-URA3-P_GAP1_-ERG10-T_ENO1_ (FAO1-P_GAP1_-ERG10-T_ENO1_ in short).

**Construction of Ts-ERG9-gda-URA3-20-20**

The Squalene synthase gene (*ERG9*) was amplified from *C. tropicalis* ATCC 20336 genomic DNA using PCR using primers ERG9-F and ERG9-R, and ligated into the plasmid vector pMD 19-T Simple to yield plasmid Ts-ERG9. Then, inverse PCR with primers rERG9-F and rERG9-R was used to eliminate the middle region of *ERG9* (1043 bp). The resulting PCR product (ERG9-Ts-ERG9) was digested with *Pst*I and *Xba*I and ligated to gda324-URA3-20-20 yielding plasmid Ts-ERG9-gda-URA3-20-20. Disruption cassette for one *ERG9* allele was isolated from plasmid Ts-ERG9-gda-URA3-20-20 by PCR using primers ERG9-F and ERG9-R.

**Construction of POX5-PZE120-01 and POX5-PZE120-03-2**

The plasmid PZE120-01 was digested with *Not*I to generate the P_FBA1_-ZSS1-T_ADH2_-T_synth7_-ERG20-P_GAP1_ cassette. After blunting its sticky ends using *pfu* DNA polymerase, the cassette was inserted into plasmid Ts-POX5-gda-URA3-20-20 that had been digested with *Xba*I and *Eco*RI, and had its sticky ends blunted, yielding plasmid Ts-POX5-gda-URA3-P_FBA1_-ZSS1-T_ADH2_-T_synth7_-ERG20-P_GAP1_ (POX5**-**PZE120-01 in short). This plasmid was digested with *Mlu*I to generate the POX5-gda-URA3-P_FBA1_-ZSS1-T_ADH2_-T_synth7_-ERG20-P_GAP1_-POX5 cassette. A similar strategy was used to construct plasmid Ts-POX5-gda-URA3-P_FBA1_-ZSS1-PTS1-T_ADH2_-T_synth7_-PTS1-ERG20-P_GAP1_ (POX5**-**PZE120-03-2 in short).

**Construction of PIE112-01-2 and PIE112-03-2**

The ORF of the isopentenyl diphosphate isomerase gene (*IDI1*) and mevalonate kinase (*ERG12*) were amplified from the *C. tropicalis* ATCC 20336 genome using PCR with primers IDI-F and IDI-R, and ERG12-F and ERG12-R, respectively. The *IDI1* gene was inserted into *Sal*I and *Nhe*I digested PBRP-01 to generate plasmid Ts-T_PGK1_-CtcarB-P_FBA1_-P_GAP1_-IDI1-T_ENO1_ (PIE112-01-1 in short). The *ERG20* gene was inserted into *Spe*I and *Xho*I digested PIE112-01-1 to generate plasmid Ts-T_PGK1_-ERG12-P_FBA1_-P_GAP1_-IDI1-T_ENO1_ (PIE112-01-2 in short). A similar strategy was used to construct plasmid Ts-T_PGK1_-PTS1-ERG12-P_FBA1_-P_GAP1_-IDI1-PTS1-T_ENO1_ (PIE112-03-2 in short). The fusion-protein genes IDI1-PTS1 and ERG12-PTS1 were amplified from the *C. tropicalis* ATCC 20336 genome using PCR with primers IDI-F and IDI-R2, and ERG12-F and ERG12-R2, respectively.

**Construction of PEE819-01-2 and PEE819-03-2**

The ORF of the mevalonate diphosphate decarboxylase gene (*ERG19*) and phosphomevalonate kinase (*ERG8*) were amplified from the *C. tropicalis* ATCC 20336 genome using PCR with primers ERG19-F and ERG19-R, and ERG8-F and ERG8-R, respectively. The *ERG19* gene was inserted into *Sac*I and *Eco*RI digested PZE120-01 to generate plasmid Ts-P_FBA1_-ERG19-T_ADH2_-T_synth7_-ERG20-P_GAP1_ (PEE819-01-1 in short). The *ERG8* gene was inserted into *Spe*I and *KpnI* digested PEE819-01-1 to generate plasmid Ts-P_FBA1_-ERG19-T_ADH2_-T_synth7_-ERG8-P_GAP1_ (PEE819-01-2 in short). A similar strategy was used to construct plasmid Ts-P_FBA1_-ERG19-PTS1-T_ADH2_-T_synth7_-PTS1-ERG8-P_GAP1_ (PEE819-03-2 in short). The fusion-protein genes ERG19-PTS1 and ERG8-PTS1 were amplified from the *C. tropicalis* ATCC 20336 genome using PCR with primers ERG19-F and ERG19-R2, and ERG8-F and ERG8-R2, respectively.

**Construction of ALD1-PIEEE819121-01-2 and ALD1-PIEEE819121-03-2**

The plasmid PEE819-01-2 was digested with *Not*I to generate the P_FBA1_-ERG19-T_ADH2_-T_synth7_-ERG20-P_GAP1_ cassette. After blunting its sticky ends using *pfu* DNA polymerase, the cassette was inserted into plasmid PIE112-01-2 that had been digested with *Not*I, and had its sticky ends blunted, yielding plasmid Ts-T_PGK1_-ERG12-P_FBA1_-P_GAP1_-ERG8-T_synth7_-T_ADH2_*-ERG19*-*P_FBA1_*-P_GAP1_-IDI1-T_ENO1_ (PIEEE819121-01-2 in short). This plasmid was digested with *Mlu*I to generate the T_PGK1_-ERG12-P_FBA1_-P_GAP1_-ERG8-T_synth7_-T_ADH2_*-ERG19*-*P_FBA1_*-P_GAP1_-IDI1-T_ENO1_ cassette, and inserted into Ts-ALD1-gda-URA3-20-20 at its *Eco*RI site by ClonExpress® II One Step Cloning Kit, yielding plasmid Ts-ALD1-gda-URA3-T_PGK1_-ERG12-P_FBA1_-P_GAP1_-ERG8-T_synth7_-T_ADH2_*-ERG19*-*P_FBA1_*-P_GAP1_-IDI1-T_ENO1_ (ALD1-PIEEE819121-01-2 in short). A similar strategy was used for the construction of plasmid Ts-ALD1-gda-URA3-T_PGK1_-PTS1-ERG12-P_FBA1_-P_GAP1_-ERG8-PTS1-T_synth7_-T_ADH2_-PTS1-ERG19-P_FBA1_-P_GAP1_-IDI1-PTS1-T_ENO1_ (ALD1-PIEEE819121-03-2 in short). The plasmids were digested with *Mlu*I respectively to generate the ALD1-gda-URA3-T_PGK1_-ERG12-P_FBA1_-P_GAP1_-ERG8-T_synth7_-T_ADH2_-ERG19-P_FBA1_-P_GAP1_-IDI1-T_ENO1_-ALD1 and ALD1-gda-URA3-T_PGK1_-PTS1-ERG12-P_FBA1_-P_GAP1_-ERG8-PTS1-T_synth7_-T_ADH2_-PTS1-ERG19-P_FBA1_-P_GAP1_-IDI1-PTS1-T_ENO1_-ALD1.

**Construction of PEE1013-01-2 and PEE1013-03-2**

The ORF of the hydroxymethylglutaryl-CoA synthase gene (*ERG13*) was amplified from the *C. tropicalis* ATCC 20336 genome using PCR with primers ERG13-F and ERG13-R, and inserted into *Spe*I and *Xho*I digested PEE1013-01-1 to generate plasmid Ts-T_PGK1_-ERG13-P_FBA1_-P_GAP1_-ERG10-T_ENO1_ (PEE1013-01-2 in short). A similar strategy was used for the construction of plasmid Ts-T_PGK1_-PTS1-ERG13-P_FBA1_-P_GAP1_-ERG10-PTS1-T_ENO1_ (PEE1013-03-2 in short). The fusion-protein genes ERG10-PTS1 and ERG13-PTS1 were amplified from the *C. tropicalis* ATCC 20336 genome using PCR with primers ERG10-F and ERG10-R2, and ERG13-F and ERG13-R2, respectively.

**Construction of FAO1-PEEt10131-01-2 and FAO1-PEEt10131-03-2**

The T_synth7_-tHMGR-P_GAP1_ cassette was inserted into plasmid PEE1013-01-2 that had been digested with *Not*I, and had its sticky ends blunted, yielding plasmid Ts-T_PGK1_-ERG13-P_FBA1_-P_GAP1_-tHMGR-T_synth7_-P_GAP1_-ERG10-T_ENO1_ (PEEt10131-01-2 in short). This plasmid was digested with *Mlu*I to generate the T_PGK1_-ERG13-P_FBA1_-P_GAP1_-tHMGR-T_synth7_-P_GAP1_-ERG10-T_ENO1_ cassette, and inserted into Ts-FAO1-gda-URA3-20-20 at its *Eco*RI site by ClonExpress® II One Step Cloning Kit, yielding plasmid Ts-FAO1-gda-URA3-T_PGK1_-ERG13-P_FBA1_-P_GAP1_-tHMGR-T_synth7_-P_GAP1_-ERG10-T_ENO1_ (FAO1-PEEt10131-01-2 in short). A similar strategy was used to construct plasmid Ts-FAO1-gda-URA3-T_PGK1_-PTS1-ERG13-P_FBA1_-P_GAP1_-tHMGR-PTS1-T_synth7_-P_GAP1_-ERG10-PTS1-T_ENO1_ (FAO1-PEEt10131-03-2 in short).

**Construction of DLD1a-PEEt10131-01, DLD1a-PIEEE819121-01-2 and DLD1a-PZE120-01**

The D-lactate dehydrogenase (*DLD1a*, GenBank: RCK67597.1) was amplified from *C. tropicalis* ATCC 20336 genomic DNA using PCR using primers DLD1-F1 and DLD1-R1, and ligated into the plasmid vector pMD 19-T Simple to yield plasmid Ts- DLD1a. Then, inverse PCR with primers rDLD1-F1 and rDLD1-R1 was used to eliminate the middle region of DLD1a (approximately 402 bp). The resulting PCR product (DLD1a-Ts-DLD1a) was digested with *Pst*I and *Xba*I and ligated to gda324-URA3-20-20 cassettes yielding plasmid Ts-DLD1a-gda-URA3-20-20.

The plasmid Ts-DLD1a-gda-URA3-T_PGK1_-ERG13-P_FBA1_-P_GAP1_-tHMGR-T_synth7_-P_GAP1_-ERG10-T_ENO1_ (DLD1a-PEEt10131-01 in short), Ts-DLD1a-gda-URA3-T_PGK1_-ERG12-P_FBA1_-P_GAP1_-ERG8-T_synth7_-T_ADH2_-ERG19-P_FBA1_-P_GAP1_-IDI1-T_ENO1_ (DLD1a-PIEEE819121-01-2 in short) and Ts-DLD1a-gda-URA3-P_FBA1_-ZSS1-T_ADH2_-T_synth7_-ERG20-P_GAP1_ (DLD1a**-**PZE120-01 in short) were constructed using the same method above. The plasmids were digested with *Mlu*I respectively, to generate the gene expression cassettes (cassette 1, DLD1a-gda-URA3-T_PGK1_-ERG13-P_FBA1_-P_GAP1_-tHMGR-T_synth7_-P_GAP1_-ERG10-T_ENO1_-DLD1a; cassette 2, DLD1a-gda-URA3-T_PGK1_-ERG12-P_FBA1_-P_GAP1_-ERG8-T_synth7_-T_ADH2_-ERG19-P_FBA1_-P_GAP1_-IDI1-T_ENO1_-DLD1a and cassette 3, DLD1a-gda-URA3-P_FBA1_-ZSS1-T_ADH2_-T_synth7_-ERG20-P_GAP1_-DLD1a).

**Construction of DLD1a**-**PFBA1-ZSS1-TADH2**

The P_FBA1_-ZSS1-T_ADH2_ cassette from plasmid PZE120-01 was amplified using PCR with primers SB-F and SB-R and then inserted into Ts-DLD1a-gda-URA3-20-20 at its *Eco*RI site by ClonExpress® II One Step Cloning Kit, yielding plasmid Ts-DLD1a-gda-URA3-P_FBA1_-ZSS1-T_ADH2_ (DLD1a-P_FBA1_-ZSS1-T_ADH2_ in short). This plasmid was digested with *Mlu*I to generate the DLD1a-gda-URA3-P_FBA1_-ZSS1-T_ADH2_-DLD1a cassette.

**Construction of LPP2**-**P_GAP1_-ZSS1-T_synth7_ and DLD1b**-**P_GAP1_-ZSS1-T_synth7_**

The lipid phosphate phosphatase (*LPP2,* GenBank: RCK57438.1) was amplified from *C. tropicalis* ATCC 20336 genomic DNA by PCR using primers LPP2-F and LPP2-R, and ligated into the plasmid vector pMD 19-T Simple to yield plasmid Ts- LPP2. Then, inverse PCR with primers rLPP2-F and rLPP2-R was used to eliminate the middle region of LPP2 (approximately 90 bp). The resulting PCR product (LPP2-Ts-LPP2) was digested with *Pst*I and *Xba*I and ligated to gda324-URA3-20-20 cassettes yielding plasmid Ts-LPP2-gda-URA3-20-20.

The D-lactate dehydrogenase (*DLD1b,* GenBank: RCK60940.1) was amplified from *C. tropicalis* ATCC 2033 genomic DNA using PCR using primers DLD1-F2 and DLD1-R2, and ligated into the plasmid vector pMD 19-T Simple to yield plasmid Ts- DLD1b. Then, inverse PCR with primers rDLD1-F2 and rDLD1-R2 was used to eliminate the middle region of DLD1b (approximately 158 bp). The resulting PCR product (DLD1b-Ts-DLD1b) was digested with *Pst*I and *Xba*I and ligated to gda324-URA3-20-20 cassettes yielding plasmid Ts-DLD1b-gda-URA3-20-20.

The ORF of *ZSS1* from plasmid Ts-*ZSS1* was amplified using PCR with primers ZSS1-F2 and ZSS1-R2, and then inserted into *Spe*I and *Kpn*I digested PZE120-01 to generate plasmid Ts-P_FBA1_-ZSS1-T_ADH2_-T_synth7_-ZSS1-P_GAP1_. The P_GAP1_-ZSS1-T_synth7_ cassette from plasmid Ts-P_FBA1_-ZSS1-T_ADH2_-T_synth7_-ZSS1-P_GAP1_ was amplified using PCR with primers St-F and St-R, and inserted into Ts-LPP2-gda-URA3-20-20 at its *Eco*RI site to generate plasmid Ts-LPP2-gda-URA3-P_GAP1_-ZSS1-T_synth7_ (LPP2-P_GAP1_-ZSS1-T_synth7_ in short). A similar strategy was used to construct plasmid Ts-DLD1b-gda-URA3-P_GAP1_-ZSS1-T_synth7_ (DLD1b-P_GAP1_-ZSS1-T_synth7_ in short).

**Construction of DLD1b-PZH-01 and DLD1b-PZH-02**

The *tHMGR* gene was amplified from the *C. tropicalis* ATCC 20336 genome using PCR with primers tHMGR-F and tHMGR-R, and inserted into Ts-P_FBA1_-ZSS1-T_ADH2_-T_synth7_-ZSS1-P_GAP1_ at its *Eco*RI and *Sac*I sites, yielding plasmid Ts-P_FBA1_-tHMGR-T_ADH2_-T_synth7_-ZSS1-P_GAP1_ (PZH-01 in short).

The ORF of the NADH-dependent HMG-CoA reductase (*NADH-HMGR*) from *Silicibacter pomeroyi* was codon-optimized and chemically synthesized (Sequence S2). The optimized *NADH-HMGR* gene was inserted into Ts-P_FBA1_-ZSS1-T_ADH2_-T_synth7_-ZSS1-P_GAP1_ at its *Eco*RI and *Sac*I sites, yielding plasmid Ts-P_FBA1_-NADH-HMGR-T_ADH2_-T_synth7_-ZSS1-P_GAP1_ (PZH-02 in short).

The plasmid PZH-01 was digested with *Not*I to generate the P_FBA1_-tHMGR-T_ADH2_-T_synth7_-ZSS1-P_GAP1_ cassette. After blunting its sticky ends using *pfu* DNA polymerase, the cassette was inserted into plasmid Ts-DLD1b-gda-URA3-20-20 that had been digested with *Xba*I and *Eco*RI, and had its sticky ends blunted, yielding plasmid Ts-DLD1b-gda-URA3-P_FBA1_-tHMGR-T_ADH2_-T_synth7_-ZSS1-P_GAP1_ (DLD1b**-**PZH-01 in short). A similar strategy was used for the construction of plasmid Ts-DLD1b-gda-URA3-P_FBA1_-NADH-HMGR-T_ADH2_-T_synth7_-ZSS1-P_GAP1_ (DLD1b**-**PZH-02 in short)

**Construction of DLD1b-PZEH-01**

The optimized *NADH-HMGR* gene was inserted into *Spe*I and *Xho*I digested PEE1013-01-1 to generate plasmid Ts-T_PGK1_-NADH-HMGR-P_FBA1_-P_GAP1_-ERG10-T_ENO1_ (PEH10R-01 in short). The T_synth7_-ZSS1-P_GAP1_ cassette was inserted into plasmid PEH10R-01 that had been digested with *Not*I, and had its sticky ends blunted, yielding plasmid Ts-T_PGK1_-NADH-HMGR-P_FBA1_-P_GAP1_-ZSS1-T_synth7_-P_GAP1_-ERG10-T_ENO1_ (PZEH-01 in short). This plasmid was digested with *Mlu*I to generate the T_PGK1_-NADH-HMGR-P_FBA1_-P_GAP1_-ZSS1-T_synth7_-P_GAP1_-ERG10-T_ENO1_ cassette, and inserted into Ts-DLD1b-gda-URA3-20-20 at its *Eco*RI site to generate plasmid Ts-DLD1b-gda-URA3-T_PGK1_-NADH-HMGR-P_FBA1_-P_GAP1_-ZSS1-T_synth7_-P_GAP1_-ERG10-T_ENO1_ (DLD1b-PZEH-01 in short).

**Construction of sgRNA expression cassettes**

The sgRNA expression cassettes were constructed as described in our previous work [[4](#_ENREF_4)]. The 20 nt guide sequences used in this study were selected using the bioinformatics tool sgRNACas9 [[5](#_ENREF_5)], and the fusion sgRNA “HH ribozyme-N20 (N indicates any base) target sequence-sgRNA scaffold-HDV ribozyme” was chemically synthesized (Sequence S3). The fusion fragments were expressed under the control of the *GAP1* promoter and terminator, yielding plasmids Ts-P_GAP1_-HH-sgRNA-FAO1-HDV-T_GAP1_, Ts-P_GAP1_-HH-sgRNA-ALD1-HDV-T_GAP1_, Ts-P_GAP1_-HH-sgRNA-POX5-HDV-T_GAP1_, Ts-P_GAP1_-HH-sgRNA-DLD1a-HDV-T_GAP1_, Ts-P_GAP1_-HH-sgRNA-DLD1b-HDV-T_GAP1_ and Ts-P_GAP1_-HH-sgRNA-LPP2-HDV-T_GAP1_, respectively. The sgRNA expression cassettes were isolated from plasmids by PCR using primers GAP1-F and GAP1-R, respectively.

**References**

1. L. H. Zhang, H. B. Zhang, Y. F. Liu, J. Y. Zhou, W. Shen, L. M. Liu, Q. Li and X. Z. Chen, *Biotechnology and bioengineering*, 2020, **117**, 531-542.

2. L. H. Zhang, X. Z. Chen, Z. Chen, Z. Z. Wang, S. Jiang, L. Li, M. Potter, W. Shen and Y. Fan, *Applied microbiology and biotechnology*, 2016, **100**, 9567-9580.

3. K. A. Curran, N. J. Morse, K. A. Markham, A. M. Wagman, A. Gupta and H. S. Alper, *ACS synthetic biology*, 2015, **4**, 824-832.

4. L. Zhang, H. Zhang, Y. Liu, J. Zhou, W. Shen, L. Liu, Q. Li and X. Chen, *Biotechnology and bioengineering*, 2020, **117**, 531-542.

5. S. Xie, B. Shen, C. Zhang, X. Huang and Y. Zhang, *PloS one*, 2014, **9**, e100448.
