# Supplementary material for: Engineering the oleaginous yeast Candida tropicalis for α-humulene overproduction
Source: Biotechnol Biofuels Bioprod. 2022 May 26;15:59. doi: 10.1186/s13068-022-02160-8 (PMC9137083; doi:10.1186/s13068-022-02160-8)
Supplement: Supplementary file 2 — Additional file 2: Fig S1. GC-MS analysis of α-humulene from the dodecane of the cultures in engineered C. tropicalis. Fig S2 Bioinformatic analysis of HMGR1 protein from C. tropicalis ATCC20336. Fig S3 Transcription levels of the ERG9 gene in HC02 and HC07 strains. Fig S4 Confirmation of the synthetic terminator Tsynth7 using GFP as a reporter. [file 13068_2022_2160_MOESM2_ESM.docx]

**Additional Figure captions**

**Fig S1.** GC-MS analysis of α-humulene from the dodecane of the cultures in engineered *C. tropicalis*.

**Fig S2.** Bioinformatic analysis of *HMGR1* protein from *C. tropicalis* ATCC20336.

**Fig S3.** Transcription levels of the *ERG9* gene in HC02 and HC07 strains.

**Fig S4.** Confirmation of the synthetic terminator *T_synth7_* using GFP as a reporter.


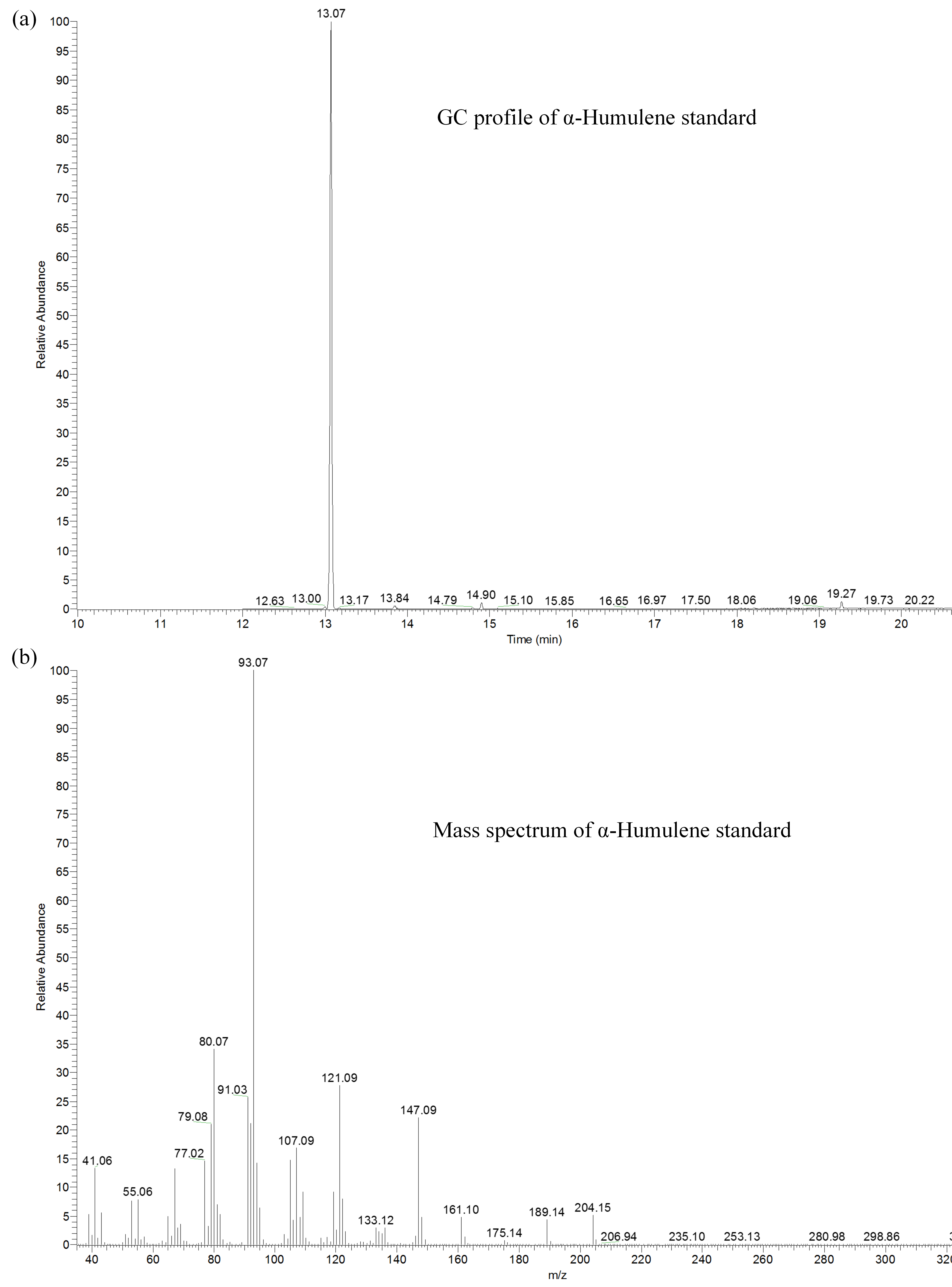

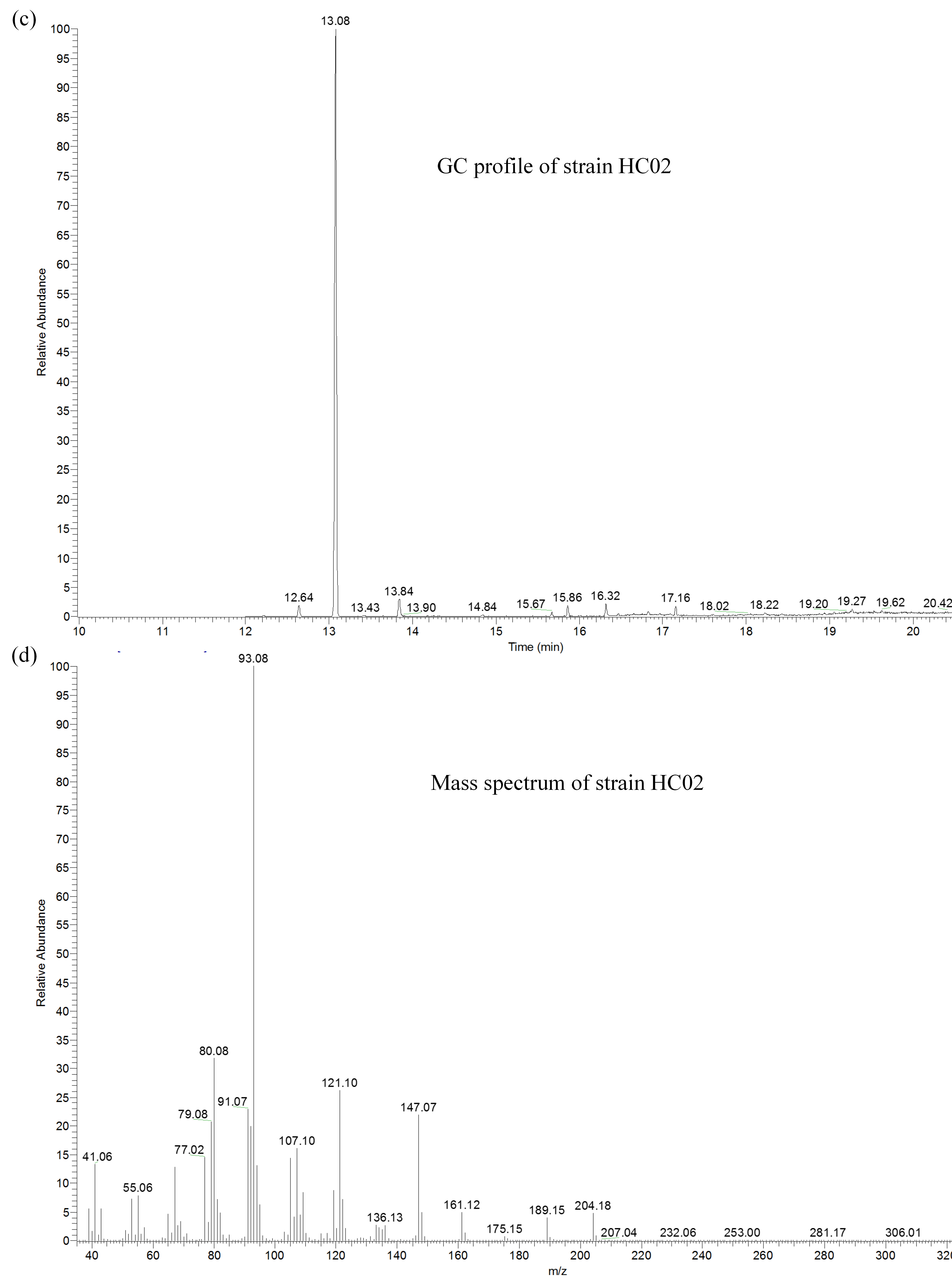

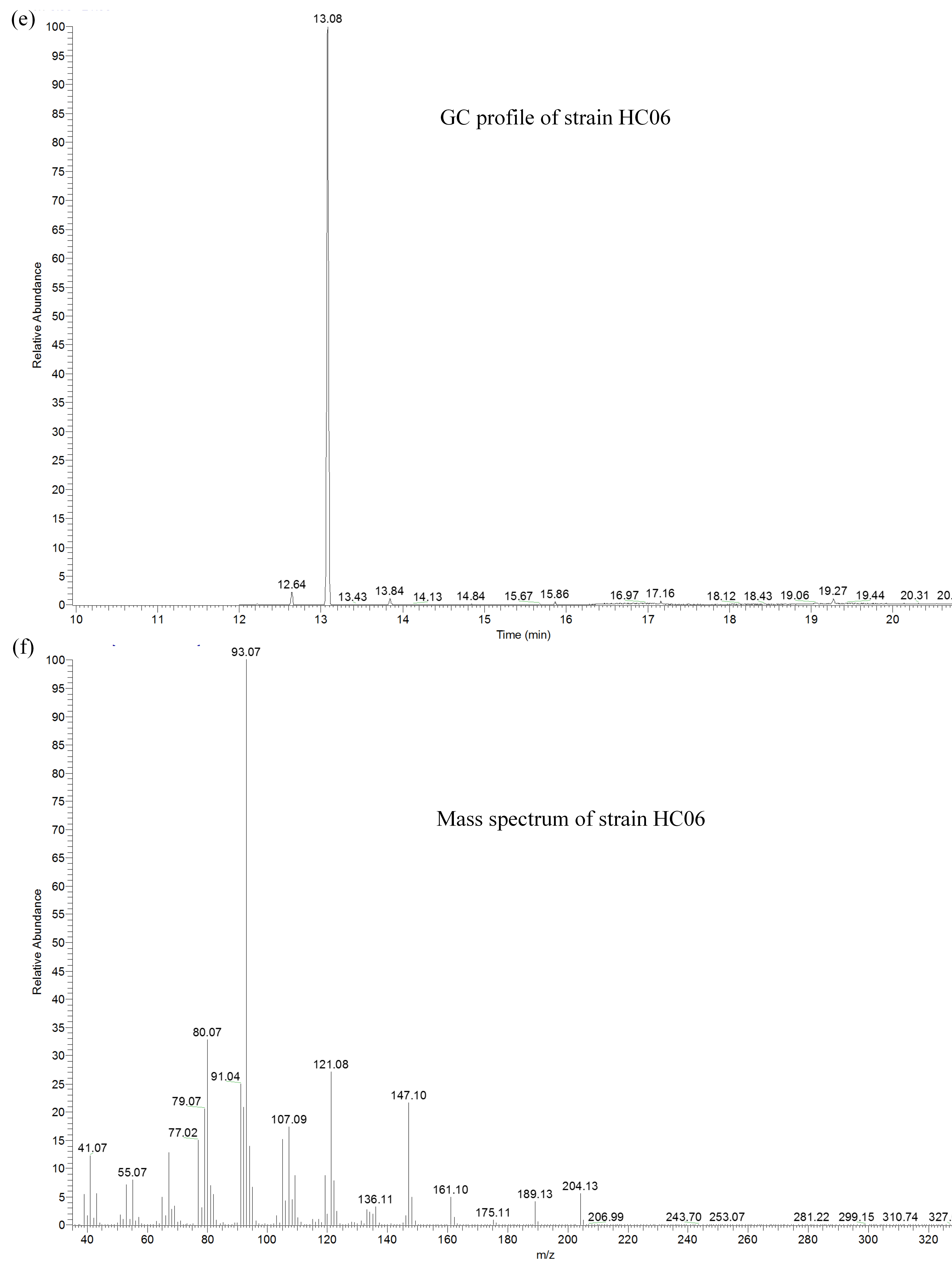

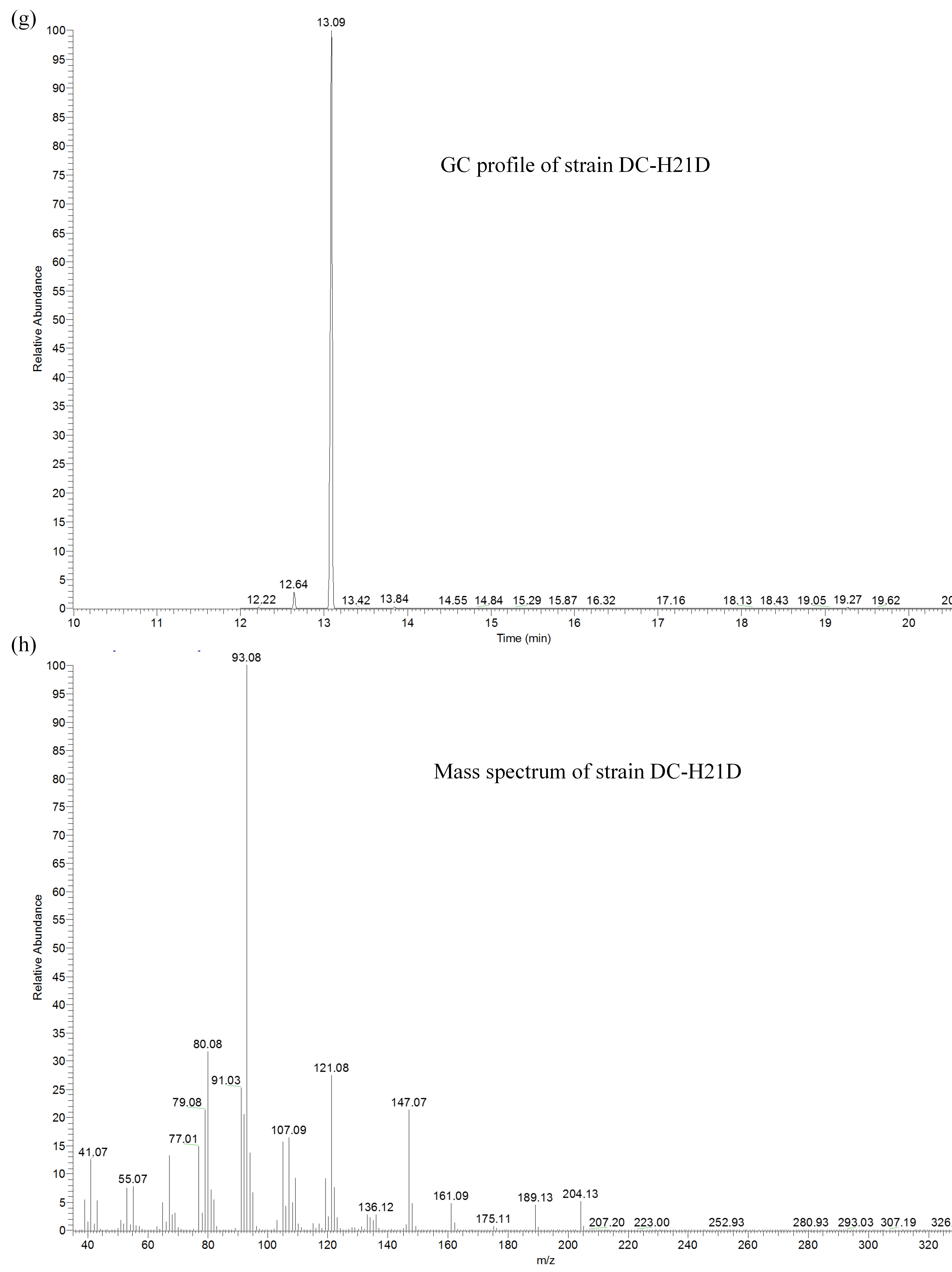


**Fig S1.** **GC-MS analysis of α-humulene from the dodecane of the cultures in engineered *C. tropicalis***. (a) GC profile of α-humulene standard. (b) Mass spectrum of α-humulene standard. (c) GC profile of α-humulene produced by HC02 strain. (d) Mass spectrum of α-humulene produced by HC02 strain. (e) GC profile of α-humulene produced by HC06 strain. (f) Mass spectrum of α-humulene produced by HC06 strain. (g) GC profile of α-humulene produced by DC-H21D strain. (h) Mass spectrum of α-humulene produced by DC-H21D strain.


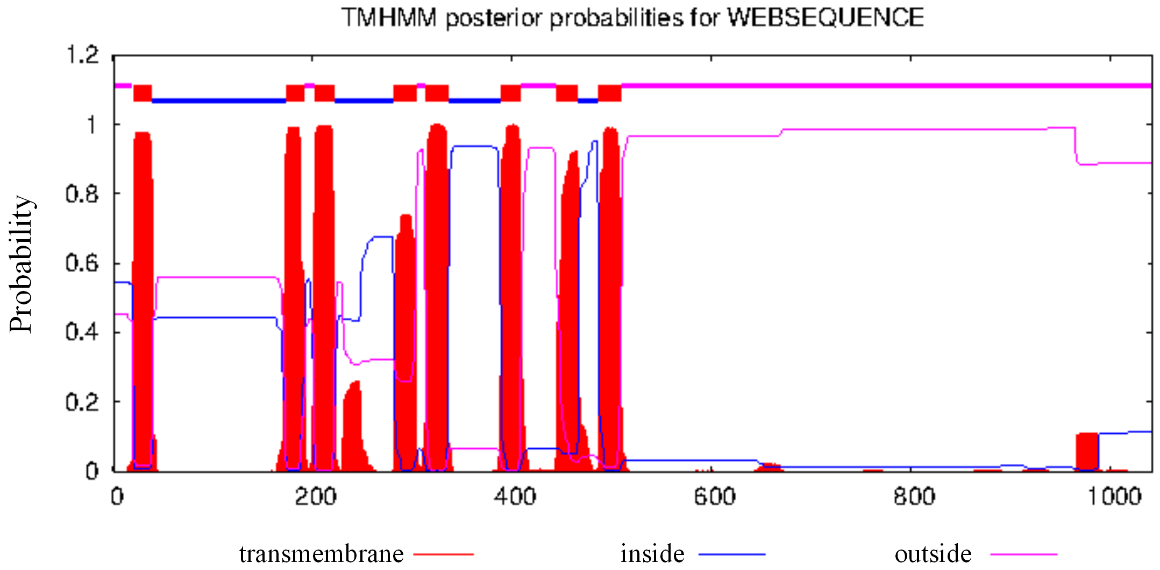


**Fig S2. Bioinformatic analysis of *HMGR1* protein from *C. tropicalis* ATCC20336.** The transmembrane possibility was demonstrated by the TMHMM tool online.


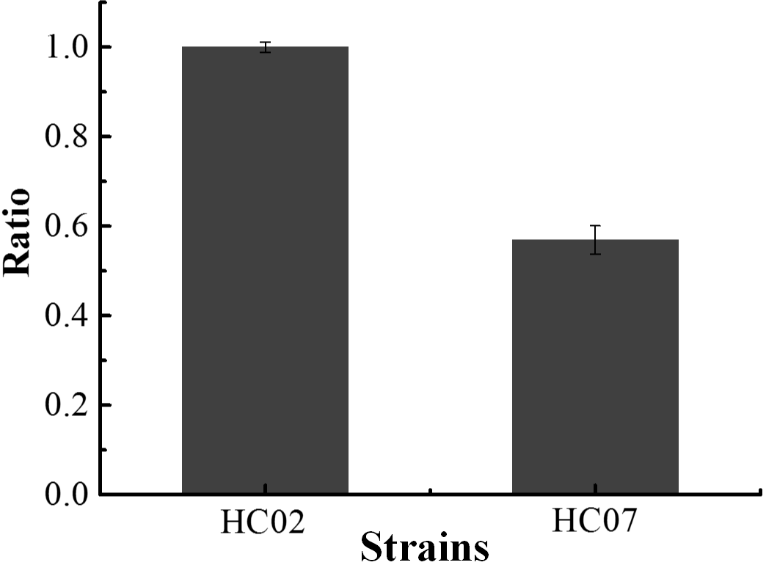


**Fig S3. Transcription levels of the *ERG9* gene in HC02 and HC07 strains.**

**Fig S4. Confirmation of the synthetic terminator *T_synth7_* using GFP as a reporter.** Contrast represents a transformant expressing *yeGFP3* under the control of the *GAP1* promoter and terminator (*C. tropicalis* 05-3); *T_synth7_* represents a transformant expressing *yeGFP3* under the control of the *GAP1* promoter and *T_synth7_* terminator (*C. tropicalis* 02).
