# Supplementary material for: Engineering the oleaginous yeast Candida tropicalis for α-humulene overproduction
Source: Biotechnol Biofuels Bioprod. 2022 May 26;15:59. doi: 10.1186/s13068-022-02160-8 (PMC9137083; doi:10.1186/s13068-022-02160-8)
Supplement: Supplementary file 3 — Additional file 3: Table S1. Primers used for plasmids construction. [file 13068_2022_2160_MOESM3_ESM.docx]

**Table S1.** Primers used for plasmids construction

| **Primer name** | **Sequence (5' to 3')** |
| --- | --- |
| SKL-F | GTATGGATGAATTGTACAAATCCAAGTTGT AACTATCCAACAAACTCTAG |
| SKL-R | CTAGAGTTTGTTGGATAGTTACAACTTGGATTTGTACAATTCATCCATAC |
| ZSS-F | CAATTAAAGAATTTAAACAGCCACCATGGAAAGACAATCC |
| ZSS-R | TAATTCTTCACCTTTAGACATAGAACCACCACCACCAATCAAGAAGGATTCAACAAA |
| PEX3-F | CAATTAAAGAATTTAAACAATGGCTATATTCTCATCATTAAG |
| PEX3-R | GCTTCACCTTTAGAAACCATAGAACCACCACCACCTTCAAAGTTGGAATATATACCG |
| TSYN7-F | GATGAATTGTACAAATAATATATAACTGTCTAGAAATAAATTTTTTCAAATCTTGCCTTTCGACATC |
| TSYN7-R | GATGTCGAAAGGCAAGATTTGAAAAAATTTATTTCTAGACAGTTATATATTATTTGTACAATTCATC |
| T7TA2-F | GGACTAGTTATATAACTGTCTAGAAATAAATTTTTTCAAAGGTGGCACAAAGAATGAAGG |
| T7TA2-R | TGCGGCCGCCGAGCTCGTGTACTGAAATTGGTGGGGGAAAA |
| PFBA-F1 | ATTTGCGGCCGCTACGATAACCCATTGATTTCT |
| PFBA-R1 | CGGAATTCTGTTGGATGATTTGTAGAT |
| PGAP-F1 | GCTCTAGAGCGGCCGCAACGTGGTATGGTTGTAAGAAAC |
| PGAP-R1 | GCGTCGACTGTTTAAATTCTTTAATTG |
| ERG20-F | TCAATTAAAGAATTTAAACAGGTACCATGTCTGATAAAGCAGCCGCTAG |
| ERG20-R | CTGCCGTTCGACGATTCGGACTAGTCTATTTGGATCTCTTGTAAACCT |
| ZSS1-F | ATCTACAAATCATCCAACA GCCACCATGGAAAGACAATCC |
| ZSS1-R | CCACCAATTTCAGTACACTTACAACTTGGAAATCAAGAAGGATTCAACAAA |
| ERG20-R2 | ATTTCTAGACAGTTATATATTACAACTTGGATCTTCTACCTCTACCCAATTTGGATCTCTTGTAAACCT |
| POX5-F | CGACGCGTCATGAAATAAGCCTAAACTCAC |
| POX5-R | CGACGCGTCACTTGTCATCTGATTTACAGC |
| rPOX5-F | AACTGCAGCACCAATCAAAGCCGAGTA |
| rPOX5-R | GCTCTAGAcgTCTTACAACGGTTTCGGTA |
| SB-F | GAAGACCAGACGTGATTAGG  TACGATAACCCATTGATTTCT |
| SB-R | TCGTTGTCGGTGTCAAGGTGCGGTGGCACAAAGAATGAAGG |
| tHMG-F1 | TCAATTAAAGAATTTAAACAGCCACCATGGCCCGTTTCCAAGTTTCAGCTAC |
| tHMG-R2 | TATTTCTAGACAGTTATATA TTA CAACTTGGATCTTCTACCTCTACCCAA TGACTTTATACATGTAACTGAT |
| St-F | TCGTTGTCGGTGTCAAGGTGC  ACGTGGTATGGTTGTAAGAAAC |
| St-R | GAAGACCAGACGTGATTAGG  CCTTCATTCT TTGTGCCACC |
| FAO1-F1 | CGACGCGTGGGAAACCGTCTACAACACC |
| FAO1-R1 | CGACGCGTGTAACGCAACAAGTCTCGTCT |
| rFAO1-F1 | AACTGCAGCGTCTTAAGACAGGCTGACCA |
| rFAO1-R1 | GCTCTAGACTTTGCTGCTGATGAAGCATA |
| TPGK1-F | CCGCTCGAGATCAGAAATATTTATTCTAAT |
| TPGK1-R | GCTCTAGACGACGCGTTCGTTGTCGGTGTCAAGGTGC |
| HMG-F | TCAATTAAAGAATTTAAACAGCCACCATGCTAAGTTTCATCACCGAAG |
| HMG-R | TATTTCTAGACAGTTATATATTATGACTTTATACATGTAACTGAT |
| TENO1-R | GAAGACCAGACGTGATTAG |
| TPGK1-F | CCGCTCGAGATCAGAAATATTTATTCTAAT |
| TPGK1-R | GCTCTAGACGACGCGTTCGTTGTCGGTGTCAAGGTGC |
| ERG10-F | TCAATTAAAGAATTTAAACA GCCACC ATGGCTCTCCCACCAGTCTACATC |
| ERG10-R | TACCTCTTTG CAGAAAAC TTACAACTTGGCGTCGGCGTC |
| ERG10-R2 | TACCTCTTTG CAGAAAAC TTA CAACTTGGATCTTCTACCTCTACCCAA AATCTTTTCA ATAACGACAG CAGAC |
| ERG9-F | ACGCGTATCCTCTCGTCATGTGCTTCG |
| ERG9-R | ACGCGTGTGACCAACTGACTCGCTCT |
| rERG9-F | GCTCTAGACTGCGAAATGTTGTACCCA |
| rERG9-R | AACTGCACGTAACATCTGATCAAGTCGG |
| IDI-F | TCAATTAAAGAATTTAAACA GCCACC ATGTCATCAGAATACGCTAA**G**CTA GT |
| IDI-R | TACCTCTTTG CAGAAAAC TTA AAGTAATCTATGTATCTCAGTAT |
| IDI-R2 | TACCTCTTTGCAGAAAAC TTA CAACTTGGATCTTCTACCTCTACCCAA AAGTAATCTATGTATCTCAG TAT |
| ERG12-F | ATCTACAAATCATCCAACA GCCACC ATGTCAGTCTCGCCTTTCGTTGT |
| ERG12-R | TAGAATAAATATTTCTGAT TCACCAGAACTTCCATTCTTTAACGTTTATCAT |
| ERG12-R2 | TAGAATAAATATTTCTGAT TTACAACTTG GATCTTCTAC CTCTACCCAA CCAGAACTTCCATTCTTTAACGTTTATCAT |
| ERG19-F | ATCTACAAATCATCCAACA GCCACC ATGTATTCAGCTTCAGTCACAG |
| ERG19-R | CCACCAATTTCAGTACAC TTAGTTTGGTAACCCACTTTCGTTG |
| ERG19-R2 | CCACCAATTTCAGTACAC TTA CAACTTGGATCTTCTACCTCTACCCAA GTTTGGTAACCCACTTTCGTTG |
| ERG8-F | TCAATTAAAGAATTTAAACA GCCACC ATGTCAAAAGCTTTTAGTGCCCCTGGT |
| ERG8-R | TATTTCTAGA CAGTTATATA TTATAAACCGATATAGTCCTCCCAG |
| ERG8-R2 | TATTTCTAGA CAGTTATATA TTACAACTTG GATCTTCTAC CTCTACCCAA TAAACCGATATAGTCCTCCCAG |
| ALD-F1 | CGACGCGT~~T~~CCCCACCAACATCAGCCAGGAT |
| ALD-R1 | CGACGCGT~~CGT~~CAACATTGGGGAAGAACTCT |
| rALD-F1 | AACTGCAGAATCTTTGCTCAAGGCGTCAC |
| rALD-R1 | GCTCTAGACGCTTTCAACTATCCATTGTTCG |
| ERG13-F | ATCTACAAATCATCCAACA GCCACC ATGACTAACGCACCACAAAACAT |
| ERG13-R | TAGAATAAATATTTCTGAT TTATTCCTTTATAGCATAACTTCTTCT |
| ERG13-R2 | TAGAATAAATATTTCTGAT TTACAACTTG GATCTTCTAC CTCTACCCAA TTCCTTTATAGCATAACTTCTTCT |
| DLD1-F1 | CGACGCGTCGAAGTTCAGGACGACTCACC |
| DLD1-R1 | CGACGCGTTGGCAAGTATCTTCTCTTACCC |
| rDLD1-F1 | AACTGCAGTGAGTATATCACTGTTGCCGCATT |
| rDLD1-R1 | GCTCTAGATTCATATTCAGGAAGGAAACGGAA |
| DLD1-F2 | CGACGCGTGAAGAGTACACTACGGCCACT |
| DLD1-R2 | CGACGCGTCATTCGATCAACCACGGCCTT |
| rDLD1-F2 | GCTCTAGATTAAAACACGGCAACGACCTCC |
| rDLD1-R2 | AACTGCAGAAACATGAGGTGTGATCCGTCTTGGT |
| LPP2-F | CGACGCGTTTTCGCTCTCTTCTCGTTC |
| LPP2-R | CGACGCGTCGCTTATGTTTTGTACACGGAT |
| rLPP2-F | GCTCTAGACACGGCCAGATTTCATCGAC |
| rLPP2-R | AACTGCAGCCATTGCTCCCAACAAACACAA |
| ZSS1-F2 | GG***GGTACC***GCCACCATGGAAAGACAATCC |
| ZSS1-R2 | GG***ACTAGT***CTA AATCAAGAAGGATTCAACAAA |
| tHMGR-F | ATCTACAAATCATCCAACAGCCACCATGGCCCGTTTCCAAGTTTCAGCTAC |
| tHMGR-R | CCACCAATTTCAGTACACTTATGACTTTATACATGTAACTGAT |
| GAP1-F | AACGTGGTATGGTTGTAAGAAAC |
| GAP1-R | CTGGTTTAGAAGTAGGGACTGTATG |

**Sequence S1** Codon-optimized *ZSS1*

1 GCGGCCGCTA AACTATCGGA ATTCGCCACC ATGGAAAGAC AATCCATGGC TTTGGTTGGT

61 GATAAGGAAG AAATTATTAG AAAGTCCTTC GAATACCACC CAACCGTTTG GGGTGATTAC

121 TTCATTAGAA ACTACTCCTG TTTGCCATTG GAAAAGGAAT GTATGATTAA GAGAGTTGAA

181 GAATTGAAGG ATAGAGTTAG AAACTTGTTC GAAGAAACCC ACGATGTTTT GCAAATTATG

241 ATTTTGGTTG ATTCCATTCA ATTGTTGGGT TTGGATTACC ACTTCGAAAA GGAAATTACC

301 GCTGCTTTGA GATTGATTTA CGAAGCTGAT GTTGAAAACT ACGGTTTGTA CGAAGTTTCC

361 TTGAGATTCA GATTGTTGAG ACAACACGGT TACAACTTGT CCCCTGATGT TTTCAACAAG

421 TTCAAGGATG ATAAGGGTAG ATTCTTGCCA ACCTTGAACG GTGATGCTAA GGGTTTGTTG

481 AACTTGTACA ACGCTGCTTA CTTGGGCACC CACGAAGAAA CCATTTTGGA TGAAGCTATT

541 TCCTTCACCA AGTGTCAATT GGAATCCTTG TTGGGTGAAT TGGAACAACC ATTGGCTATT

601 GAGGTTTCCT TGTTCTTGGA AACCCCATTG TACAGAAGAA CTAGAAGATT GTTGGTTAGA

661 AAGTACATTC CAATTTACCA AGAAAAGGTT ATGAGAAACG ATACCATTTT GGAATTGGCT

721 AAGTTGGATT TCAACTTGTT GCAATCCTTG CACCAAGAAG AAGTTAAGAA AATTACCATT

781 TGGTGGAACG ATTTGGCTTT GACCAAGTCC TTGAAGTTCG CTAGAGATAG AGTTGTTGAA

841 TGTTACTACT GGATTGTTGC TGTTTACTTC GAACCACAAT ACTCTAGAGC TAGAGTTATT

901 ACCTCCAAGG CTATTTCCTT GATGTCCATT ATGGATGATA TTTACGATAA CTACTCCACC

961 TTGGAAGAAT CTAGATTGTT GACCGAAGCT ATTGAAAGAT GGGAACCACA AGCTGTTGAT

1021 TGTGTTCCTG AATACTTGAA GGATTTCTAC TTGAAGTTGT TGAAGACCTA CAAGGATTTC

1081 GAAGATGAAT TGGAACCAAA CGAAAAGTAC CGTATTCCAT ACTTGCAAGA AGAAATTAAG

1141 GTTTTGTCCC GTGCTTACTT CCAAGAAGCT AAGTGGGGTG TTGAAAGATA CGTTCCTGCT

1201 TTGGAAGAAC ACTTGTTGGT CTCCTTAATT ACCGCTGGTT ACTTCGCTGT TGCTTGTGCT

1261 TCCTACGTTG GTTTGGGTGA AGATGCTACC AAGGAAACCT TCGAATGGGT TGCTTCCTCC

1321 CCAAAGATTT TGAAGTCCTG TTCCATTCAC TGTAGATTGA TGGATGATAT CACTTCCCAC

1381 CAAAGAGAAC AAGAAAGAGA TCACTTCGCT TCCACCGTTG AATCCTACAT GAAGGAACAC

1441 GGCACCTCCG CTAAGGTTGC TTGTGAAAAG TTGCAAGTTA TGGTTGAACA AAAGTGGAAG

1501 GATTTGAACG AAGAATGTTT GAGACCAACC CAAGTTGCTA GACCATTGAT TGAAATTATT

1561 TTGAACTTGT CTAGAGCTAT GGAAGATATT TACAAGCACA AGGATACCTA CACCAACTCC

1621 AACACTAGAA TGAAGGATAA CGTTTCCTTG ATTTTTGTTG AATCCTTCTT GATTTAGGAGCTC

The restriction sites introduced into the *ZSS1* gene are underlined

**Sequence S2** Codon-optimized *NADH-HMGR*

1 GAATTCGCCA CCATGACCGG TAAGACCGGT CACATTGATG GTTTGAACTC TAGAATTGAA

61 AAGATGAGAG ATTTGGATCC TGCTCAAAGA TTGGTCCGTG TTGCTGAAGC TGCTGGTTTG

121 GAACCTGAAG CTATTTCCGC TTTGGCTGGT AACGGTGCTT TGCCATTGTC CTTGGCTAAC

181 GGTATGATTG AAAACGTTAT TGGTAAGTTC GAATTGCCAT TAGGTGTTGC TACCAACTTC

241 ACCGTTAACG GTAGAGATTA CTTGATTCCA ATGGCTGTTG AAGAACCATC CGTTGTTGCT

301 GCCGCTTCCT ACATGGCTAG AATTGCTAGA GAAAACGGTG GTTTCACCGC TCACGGCACC

361 GCTCCATTGA TGCGTGCTCA AATTCAAGTT GTTGGTTTGG GTGACCCTGA AGGTGCTAGA

421 CAAAGATTGT TGGCTCACAA GGCTGCTTTC ATGGAAGCTG CTGATGCTGT TGATCCTGTT

481 TTGGTTGGTT TAGGCGGTGG TTGTAGAGAT ATTGAAGTCC ACGTTTTCAG AGATACCCCT

541 GTTGGTGCTA TGGTTGTTTT GCACTTGATT GTTGATGTTA GAGATGCTAT GGGTGCTAAC

601 ACCGTTAACA CCATGGCTGA AAGATTGGCT CCTGAAGTTG AAAGAATTGC TGGTGGCACC

661 GTTAGATTGA GAATTTTGTC CAACTTGGCT GATTTGCGTT TGGTTAGAGC TAGAGTTGAA

721 TTAGCTCCTG AAACCTTAAC CACCCAAGGT TACGATGGTG CTGATGTTGC TAGAGGTATG

781 GTTGAAGCTT GTGCTTTGGC TATTGTTGAT CCATACAGAG CTGCTACCCA CAACAAGGGT

841 ATTATGAACG GTATTGATCC TGTTGTCGTT GCTACCGGTA ACGATTGGAG AGCTATCGAG

901 GCTGGTGCTC ACGCTTACGC TGCTAGAACC GGTCACTACA CTTCCTTGAC TAGATGGGAA

961 TTGGCTAACG ATGGTAGATT GGTTGGCACC ATTGAATTGC CATTGGCTTT GGGTTTGGTT

1021 GGTGGCGCTA CCAAGACCCA CCCAACCGCT AGAGCTGCTT TGGCTTTGAT GCAAGTTGAA

1081 ACCGCTACCG AATTGGCTCA AGTTACCGCT GCTGTTGGTT TGGCTCAAAA CATGGCTGCT

1141 ATTAGAGCTT TGGCTACCGA AGGTATTCAA AGAGGTCACA TGACCTTGCA CGCTAGAAAC

1201 ATTGCTATTA TGGCTGGTGC TACCGGTGCT GATATTGATA GAGTTACTAG AGTTATTGTT

1261 GAGGCCGGCG ATGTTTCCGT TGCTAGAGCT AAGCAAGTTT TGGAAAACAC CTAGGAGCTC

The restriction sites introduced into the *NADH-HMGR* gene are underlined

**Sequence S3** Sequence of HH-N20 target sequence-sgRNA-HDV

**(A)** Sequence of HH-sgRNA1-FAO1-HDV

1 *GGTACC*TGAA TGCTGATGAG TCCGTGAGGA CGAAACGAGT AAGCTCGTC**C ATTCAAGGTG**

61 **CGTAAGGAA**G TTTTAGAGCT AGAAATAGCA AGTTAAAATA AGGCTAGTCC GTTATCAACT

121 TGAAAAAGTG GCACCGAGTC GGTGCTTTTg gccggcatgg tcccagcctc ctcgctggcg

181 ccggctgggc aacatgcttc ggcatggcga atgggac*AAG CTT*

**(B)** Sequence of HH-sgRNA1-ALD1-HDV

1 *GGTACC*ACAT CGCTGATGAG TCCGTGAGGA CGAAACGAGT AAGCTCGTC**C GATGTCGCAA**

61 **TTGCACAAG**G TTTTAGAGCT AGAAATAGCA AGTTAAAATA AGGCTAGTCC GTTATCAACT

121 TGAAAAAGTG GCACCGAGTC GGTGCTTTTg gccggcatgg tcccagcctc ctcgctggcg

181 ccggctgggc aacatgcttc ggcatggcga atgggac*AAG CTT*

**(C)** Sequence of HH-sgRNA1-POX4-HDV

1 *GGTACC*GTCC AACTGATGAG TCCGTGAGGA CGAAACGAGT AAGCTCGTC**T TGGACGAAGC**

61 **TGTCAGTGC**G TTTTAGAGCT AGAAATAGCA AGTTAAAATA AGGCTAGTCC GTTATCAACT

121 TGAAAAAGTG GCACCGAGTC GGTGCTTTTg gccggcatgg tcccagcctc ctcgctggcg

181 ccggctgggc aacatgcttc ggcatggcga atgggac*AAG CTT*

**(D)** Sequence of HH-sgRNA1-DLD1a-HDV

1 *GGTACC*ACCT GGCTGATGAG TCCGTGAGGA CGAAACGAGT AAGCTCGTC**C CAGGTATCGG**

61 **TGCTAACAT**G TTTTAGAGCT AGAAATAGCA AGTTAAAATA AGGCTAGTCC GTTATCAACT

121 TGAAAAAGTG GCACCGAGTC GGTGCTTTTg gccggcatgg tcccagcctc ctcgctggcg

181 ccggctgggc aacatgcttc ggcatggcga atgggac*AAG CTT*

**(E)** Sequence of HH-sgRNA1-DLD1b-HDV

1 *GGTACC*TTGA ACCTGATGAG TCCGTGAGGA CGAAACGAGT AAGCTCGTC**G TTCAAGTACG**

61 **GCACGATGA**G TTTTAGAGCT AGAAATAGCA AGTTAAAATA AGGCTAGTCC GTTATCAACT

121 TGAAAAAGTG GCACCGAGTC GGTGCTTTTg gccggcatgg tcccagcctc ctcgctggcg

181 ccggctgggc aacatgcttc ggcatggcga atgggac*AAG CTT*

**(F)** Sequence of HH-sgRNA1-LPP2-HDV

1 *GGTACC*GTCG CCCTGATGAG TCCGTGAGGA CGAAACGAGT AAGCTCGTC**G GCGACACAAT**

61 **TGCTTGCAA**G TTTTAGAGCT AGAAATAGCA AGTTAAAATA AGGCTAGTCC GTTATCAACT

121 TGAAAAAGTG GCACCGAGTC GGTGCTTTTg gccggcatgg tcccagcctc ctcgctggcg

181 ccggctgggc aacatgcttc ggcatggcga atgggac*AAG CTT*

The chemical base with a wavy line represents hammerhead ribozyme. The chemical base in italics are reverse compliment of the first six nucleotides in the 20 bp target sequence. The chemical base bold represents the 20 bp target sequence. The underlined chemical base represents the sgRNA scaffold. The lowercase letter represents the hepatitis delta virus ribozyme. The restriction sites introduced into the HH-N20 target sequence-sgRNA-HDV are italicized.
